# A Role for Thalamic Projection GABAergic Neurons in Circadian Responses to Light

**DOI:** 10.1523/JNEUROSCI.0112-21.2022

**Published:** 2022-12-07

**Authors:** Olivier Brock, Cigdem Gelegen, Peter Sully, Irene Salgarella, Polona Jager, Lucy Menage, Ishita Mehta, Jagoda Jęczmień-Łazur, Deyl Djama, Lauren Strother, Angelica Coculla, Anthony C. Vernon, Stephen Brickley, Philip Holland, Samuel F. Cooke, Alessio Delogu

**Affiliations:** ^1^Department of Basic and Clinical Neuroscience, Institute of Psychiatry, Psychology and Neuroscience, King's College London, London SE5 9NU, United Kingdom; ^2^Department of Life Sciences and Centre for Neurotechnology, Imperial College London, London SW7 2AZ, United Kingdom; ^3^MRC Centre for Neurodevelopmental Disorders, King's College London, London SE1 1UL, United Kingdom; ^4^Wolfson Centre for Age Related Disease, King's College London, London SE1 1UL, United Kingdom

**Keywords:** EEG, intergeniculate leaflet, melanopsin, sleep, Sox14, thalamus

## Abstract

The thalamus is an important hub for sensory information and participates in sensory perception, regulation of attention, arousal and sleep. These functions are executed primarily by glutamatergic thalamocortical neurons that extend axons to the cortex and initiate cortico-thalamocortical connectional loops. However, the thalamus also contains projection GABAergic neurons that do not extend axons toward the cortex. Here, we have harnessed recent insight into the development of the intergeniculate leaflet (IGL) and the ventral lateral geniculate nucleus (LGv) to specifically target and manipulate thalamic projection GABAergic neurons in female and male mice. Our results show that thalamic GABAergic neurons of the IGL and LGv receive retinal input from diverse classes of retinal ganglion cells (RGCs) but not from the M1 intrinsically photosensitive retinal ganglion cell (ipRGC) type. We describe the synergistic role of the photoreceptor melanopsin and the thalamic neurons of the IGL/LGv in circadian entrainment to dim light. We identify a requirement for the thalamic IGL/LGv neurons in the rapid changes in vigilance states associated with circadian light transitions.

**SIGNIFICANCE STATEMENT** The intergeniculate leaflet (IGL) and ventral lateral geniculate nucleus (LGv) are part of the extended circadian system and mediate some nonimage-forming visual functions. Here, we show that each of these structures has a thalamic (dorsal) as well as prethalamic (ventral) developmental origin. We map the retinal input to thalamus-derived cells in the IGL/LGv complex and discover that while RGC input is dominant, this is not likely to originate from M1ipRGCs. We implicate thalamic cells in the IGL/LGv in vigilance state transitions at circadian light changes and in overt behavioral entrainment to dim light, the latter exacerbated by concomitant loss of melanopsin expression.

## Introduction

GABAergic projection neurons are present at the rostroventral edges of the mouse thalamus to form the intergeniculate leaflet (IGL; Morin and Blanchard, 1999, 2001, 2005). Contiguous with the IGL, but within the largely GABAergic prethalamic territory, is the ventral lateral geniculate nucleus (LGv; Monavarfeshani et al., 2017; Moore et al., 2000; Morin and Blanchard, 2005; Sabbagh et al., 2020). Despite their largely distinct ontogeny (Vue et al., 2007; Inamura et al., 2011; Jeong et al., 2011; Suzuki-Hirano et al., 2011; Yuge et al., 2011; Delogu et al., 2012; Virolainen et al., 2012; Puelles et al., 2020), functional studies have often grouped the IGL and LGv together, based on GABA expression, anatomic proximity, and to some degree, shared patterns of connectivity.

The IGL and LGv are the source of the geniculohypothalamic tract (Harrington, 1997; Moore, 1989; Pu and Pickard, 1996; Morin and Blanchard, 1999, 2001) that enables regulation of the circadian clock in the suprachiasmatic nucleus (SCN; Harrington and Rusak, 1986; Johnson et al., 1989; Shibata and Moore, 1993; Huhman and Albers, 1994; Huhman et al., 1995, 1996; Lewandowski and Usarek, 2002; Hanna et al., 2017; Fernandez et al., 2020). The geniculohypothalamic tract is believed to be the conduit for integrated photic (Harrington and Rusak, 1989; Zhang and Rusak, 1989; Morin and Studholme, 2014b) and nonphotic (Johnson et al., 1988; Janik and Mrosovsky, 1994; Kuroda et al., 1997; Marchant et al., 1997; Maywood et al., 1997, 2002) cues that contribute to circadian entrainment to relevant external and internal variables. Retinal input is mostly from intrinsically photosensitive retinal ganglion cells (ipRGCs; Hattar et al., 2006; Güler et al., 2008; Fernandez et al., 2018; Huang et al., 2019; An et al., 2020; Beier et al., 2020), while nonphotic cues are thought to propagate via neurons of the ascending arousal system (Meyer-Bernstein and Morin, 1996; Marchant et al., 1997; Błasiak and Lewandowski, 2003; Vrang et al., 2003; Smith et al., 2015).

Neurons in the IGL/LGv were shown to participate in mood regulation via inhibitory synapses onto lateral habenula (LH) neurons (Huang et al., 2019) and to contribute to photosomnolence in mice exposed to unexpected light at night (Shi et al., 2020).

Thalamic GABAergic projection neurons are specified during embryonic development within the rostral portion of the second diencephalic prosomere (p2; Puelles and Rubenstein, 1993, 2003; Rubenstein et al., 1994; Vue et al., 2007; Kataoka and Shimogori, 2008; Martinez-Ferre and Martinez, 2012; Nakagawa, 2019; Puelles, 2019) and can be defined by expression of the transcription factor gene *Sox14* (Vue et al., 2007; Delogu et al., 2012; Virolainen et al., 2012; Sellers et al., 2014). Tangential cell migration during embryogenesis distributes thalamic GABAergic precursors from the prospective IGL, to the developing LGv and to other thalamic regions including the perihabenula (pHB; Delogu et al., 2012). Hence, the mature IGL and LGv are characterized by a heterogenous cellular composition that includes *Sox14*^+^ GABAergic thalamic neurons. While the sparse interneurons of the mouse thalamocortical nuclei also express *Sox14*, these local circuit cells have a distinctive mesencephalic origin (Jager et al., 2016, 2021).

Here, we used stereotaxic injections in the *Sox14^Cre^* mouse to enable the characterization of the thalamic component of anatomic regions with complex embryonic ontogeny. We demonstrate that circadian optogenetic stimulation of the *Sox14*^+^ neurons in the IGL/LGv is sufficient to reset circadian motor activity rhythms in the absence of other light cues. Upon cell ablation, we show that the thalamic component of the IGL/LGv plays a synergistic role with melanopsin photodetection to ensure photoentrainment to dim light and participates in the regulation of vigilance state transitions at circadian light changes. We map synaptic input to thalamic *Sox14*^+^ neurons in the IGL/LGv, which revealed specific patterns of retinal connectivity.

## Materials and Methods

### Animals

All mice were kept in the animal facilities of King's College London. The *Sox14*^*Cre*/+^ (Jager et al., 2016; MGI ID: MGI:5909921) and *Sox14*^*GFP*/+^ mouse lines (Crone et al., 2008; MGI ID: 3836003) were maintained in the C57Bl/6 background. The *Opn4^taulacZ^* mouse line (Hattar et al., 2002; MGI ID: MGI:2449781) was a mixed B6/129 background and was crossed to the *Sox14*^*Cre*/+^ or *Sox14*^*GFP*/+^ mouse lines. The *Dlx5/6^Cre^* (Monory et al., 2006; JAX stock #008199; MGI ID:3758328) and the *Rosa26^lsl-nuclearGFP^* (Mo et al., 2015; JAX stock #021039; MGI ID: 5443817) were maintained in the C57Bl/6 background. Experimental procedures were approved by the Ethical Committee for Animal Use of King's College London and were covered by a Project License under the United Kingdom Home Office Animals (Scientific Procedures) Act 1986. Mice were kept under normal housing conditions (7 A.M. lights on and 7 P.M. lights off, with food and water *ad libitum*), unless otherwise stated for behavioral experiments. All behavioral experiments were performed on adult (more than six weeks of age) male mice. Tract tracing experiments were performed using animals of both sexes.

### Generation of EnvA-pseudotyped, glycoprotein-deleted rabies virus

The EnvA-pseudotyped, glycoprotein-deleted rabies virus (ΔG-SADB19-eGFP, EnvA; abbreviated RVdG) was produced in house following an established protocol (Osakada and Callaway, 2013). The ΔG-SADB19-eGFP (generous gift from Prof. Roska, FMI, Basel, Switzerland) was amplified on BHK-SadGly-GFP cell culture (generous gift from Prof. Tripodi, LMB, Cambridge, United Kingdom) at 37°C, 3.5% CO_2_ to slow cell cycle. The BHK-EnvA cell line (generous gift from Prof. Tripodi) was used for pseudotyping the virus with EnvA envelope protein (grown at 37°C, 5% CO_2_). Filtered (Steriflip, 0.22um, Millipore) supernatant was stored at 4°C, concentrated by ultracentrifugation, resuspended in sterile PBS and stored at −80°C in single use aliquots.

### Brain stereotaxic surgeries

Briefly, mice were placed in a digital stereotaxic frame (World Precision Instruments) under 2.5% isoflurane anesthesia. For viral delivery, the skull was exposed by a midline scalp incision, and the stereotaxic frame was aligned using Bregma and λ as visual landmarks. A 33-gauge steel needle was placed above the skull and a hole drilled through the skull bone to expose the brain. Virus solutions (100–250 nl) were injected using a borosilicate glass needle (0.58 OD/ID mm, World Precision Instruments) connected to an air injector (Narishige) or a a Nanoject III (Drummond Scientific) injection system. The following general coordinates were used for IGL/LGv with litter-specific finer adjustments: from bregma AP = −2.25 mm; L = ±(2.20–2.40) mm; DV = −2.85 mm; for SCN from bregma AP = −0.5 mm; L = 0.15 mm; DV = -(5.0–5.25) mm. For the broader targeting of lateral thalamus and pretectum: from bregma AP = −2.25 mm; L = ±(2.05) mm; DV = −2.7 mm and −2.4 mm with 150nl of solution released at the two DV levels. The glass needle was left in place for an additional 8 min before being slowly removed. Following injection, skin was closed using biocompatible tissue glue (VetBond).

For optogenetics experiments, two cannulas (200 µm in core diameter; Doric Lenses) holding optical fibers were inserted and extended to the ventral edges of the dorsal part of the LGN and further fixed to the skull with dental cement. Mice were allowed to recover in a heating chamber and returned to their home cage after waking up. All mice received a subcutaneous injection with Carprofen (5 mg/kg) for postoperative analgesia.

For *in vivo* optogenetics experiments, the AAV5-EF1α-DIO-hChR2(H134R)-mCherry (Addgene plasmid #37082; Vector Core, University of North Carolina) was injected bilaterally into the IGL/LGv in three weeks old *Sox14^Cre/+^* mice. Control animals received a bilateral injection of a Cre-dependent adeno-associated virus (AAV) expressing the cyan fluorescent protein AAV1-EF1α-DIO-CFP (generated in house). For DTA-mediated cell ablation, the AAV1-EF1α-mCherry-flex-dta (Adgene plasmid #58536 Vector Core, University of North Carolina) was injected bilaterally into the IGL/LGv in three weeks old *Sox14^Cre^*^/+^ mice. Control animals received a bilateral injection of AAV1- EF1α-DIO-CFP (generated in house).

For monosynaptic tract tracing, we first injected equimolar ratio of AAV1-EF1α-Flex-TVA-mCherry (Addgene plasmid #38044 Vector Core, University of North Carolina) and AAV1-CMV-DIO-oG (codon optimized; Addgene plasmid # 74 290, Vector Core, University of North Carolina) unilaterally in the IGL/LGv, followed two weeks later by a second stereotaxic injection of the RV-dG-GFP in the IGL/LGv or SCN region. Animals were killed 7 days after the RVdG injection. For non-Cre-guided rabies tracing the following vectors were used: AAV1 Syn-H2B-GFP-TVA-oG-WPRE3 (catalog code BA-91, Charite' Viral Vector Core, Berlin, Germany) and RVdG-ChR2-Cherry (Addgene plasmid #32346; Salk Institute of Biological Studies Viral Vector Core, La Jolla).

### Brain immunohistochemistry and RNA *in situ* hybridization (ISH)

Mice were transcardially perfused with 4% paraformaldehyde (PFA) in PBS and the brains postfixed at 4°C overnight. Brains for ISH were stored in PFA for 5 days, to minimize RNA degradation, and all subsequent solutions were treated with diethyl pyrocarbonate (DEPC; AppliChem). The brains were cryoprotected in a sucrose gradient (10–20–30%), frozen on dry ice and cut on a cryostat (Leica) at 60 micrometers for IHC on floating sections or cryosectioned at 20 μm with coronal sections collected on Superfrost Ultra Plus slides (ThermoScientific) for ISH.

Immunohistochemistry was performed on floating brain sections. Primary antibodies were incubated on sections twice overnight at 4°C: chicken anti-GFP (1:10,000, Abcam, ab13970), rat anti-RFP (1:1000, 5f8-100, Chromotek), mouse anti-TH (1:1000, MAB5280, Millipore), rabbit anti-calbindin 2 (1:200, ab702, Abcam), goat anti-ChAT (1:1000, AB144P, Millipore), mouse anti-TPH (1:50, T0678, Sigma-Aldrich), goat anti-orexin A (1:1000, sc-8070, Santa Cruz Biotechnology), rabbit c-fos (1:800; ABE457 Sigma), goat anti-orexin B (1:1000, sc-8071, Santa Cruz Biotechnology), rabbit anti-MCH (1:1000, H-070-47, Phoenix). Secondary antibodies were incubated on sections for 2 h at room temperature (RT) at a 1:500 dilution. The secondary antibodies used were Alexa-conjugated goat anti-chicken Alexa-488 (A11039, ThermoFisher), goat anti-rat Alexa-568 (A11077, Invitrogen), and goat anti-mouse far red (A11036, Invitrogen), donkey anti-goat Alexa-568 (A11057, ThermoFisher), donkey anti-chicken Alexa-488 (703-545-155, Jackson ImmunoReasearch), donkey anti-mouse Alexa-647 (ab150107, Millipore), donkey anti-rabbit Alexa-647 (A31573, Invitrogen), donkey anti-rat Alexa-568 (Invitrogen). Blocking and antibody binding solutions where 7% goat serum/PBS with 0.3% Triton X-100 or 3-10% donkey serum/PBS with 1% BSA and O.5% Triton X-100. After DAPI staining (1:40,000 in 1× PBS; Life Technologies) the sections were mounted on Menzel–Glasser Superfrost Plus (J1800AMNZ, ThermoScientific) glass slides using the ProLong Gold antifade reagent (P36930, Invitrogen) mounting medium.

ISH was performed with a *Npy* antisense RNA probe transcribed *in vitro* from a cDNA template (IMAGE ID: 5683102). The probe was diluted to a final concentration of 800 ng/ml in hybridization buffer (50% formamide, 10% dextran sulfate, 1 mg/ml rRNA, 1× Denhardt's solution, 0.2 m NaCl, 10 mm Tris HCl, 5 mm NaH_2_PO_4_.2H_2_O, 1 mm Tris base, and 50 mm EDTA) and applied onto the slides, which were incubated in a humidified chamber at 65°C overnight. The slides were then washed three times for 30 min in wash buffer (50% formamide, 1× SSC, 0.1% Tween) at 65°C, two times for 30 min in MABT buffer (100 mm maleic acid, 150 mm NaCl, 0.1% Tween 20) at RT, and blocked for 2 h at RT [2% Boehringer Blocking Reagent (Roche), 20% inactivated sheep serum in MABT]. Sheep anti-DIG alkaline phosphatase conjugated antibody (Roche, 11093274910) was diluted 1:2000 in the blocking solution and incubated with the slides overnight at 4°C. This was followed by five 20-min washes in MABT and two 20-min washes in the AP buffer (100 mm Tris-HCl pH9.5, 100 mm NaCl, 50 mm MgCl2, 0.1% Tween 20). NBT/BCIP (Sigma) was diluted in the AP buffer and applied onto the slides for color reaction for 3–6 h at RT in the dark.

### Retina immunohistochemistry

The eyes were dissected, postfixed in 4% PFA overnight, and washed for at least 1 day in PBS at 4°C. The retinas were then dissected in ice cold PBS and washed again in PBS at 4°C. The retinas were then blocked in 10% normal donkey serum, 1% bovine serum albumin (BSA), 0.5% Triton X-100 in PBS for 1 h at RT. The following primary antibodies were used: goat anti-ChAT, 1:200 (Chemicon, AB144P); chicken anti-GFP, 1:5000 (Abcam, ab13970); rabbit anti-Opn4, 1:5000 (Advanced Targeting Systems, AB-N38, AB-N39); rabbit anti-UV cone opsin, 1:200 (Millipore, AB5407). The antibodies were diluted in 3% normal donkey serum, 1% BSA, 0.02% sodium azide, 0.5% Triton X-100 in PBS). The retinas were incubated in primary antibodies for 7 days at RT on a shaker. This was followed by three PBS washes, each for 30 min. The secondary antibodies used were donkey anti-goat AlexaFluor 633, 1:500 (ThermoFisher, A21082), donkey anti-goat AlexaFluor 647, 1:500 (Invitrogen, A21447), donkey anti-rabbit AlexaFluor 568, 1:500 (Invitrogen, A10042), and donkey anti-chicken AlexaFluor 488, 1:500 (Jackson ImmunoReasearch, 703-545-155), diluted in 3% NDS, and incubated with the retinas for 1 day at 4°C. The next day, two 30 min PBS washes, followed by incubation in DAPI (1:40,000 in PBS; Life Technologies) overnight at 4°C. The retinas were then washed in PBS and mounted, using the ProLong Diamond mounting medium (Invitrogen). Spacers (SLS, 24 × 24 mm, no. 15) were used on the slides to prevent the coverslips compressing the retinas.

### Optogenetic stimulation

Animals were chronically tethered to a branching fiberoptic patch cord (200-µm diameter core, 0.53 NA; Doric Lenses) attached to the implanted cannula and connected to a high-powered blue (470 nm) LED (Doric Lenses) under the control of an LED Driver (LEDRVP-2CH, Doric Lenses). LED source and patch cord were connected via an optical rotary joint allowing free movements of the animal in a circular cage. Mice were kept in constant darkness and allowed to free run at least a week before stimulation. Locomotor activity was monitored in 1-min bins using Clocklab software (Actimetrics, Inc). Light pulses (470 nm, 8 Hz, 10 ms in duration, 1 h) were generated ∼3 h or 6 h after the onset of the active phase, through Doric Neuroscience Studio software (Doric Lenses) and repeated daily at the same time of the day over 14 days. Light intensity at the cannula tip was determined to be 4.5 mV when driven at 1000 mA using a PM100D Optical Power Meter (Thorlabs).

### Light exposure protocol

Mice were single-housed in a circadian light-, air-, temperature-controlled ventilated cabinet (Phenome Technologies) monitored by Clocklab Chamber Control Software (Actimetrics, Inc). Mice were first entrained to 12/12 h light/dark cycle under standard light intensity (200 lux). Then, all subjects went through a “jet-lag” paradigm (6-h phase advance) using bright light (200 lux) lasting 14 days. Mice were then housed in constant darkness for 14 days. Following these light conditions, mice were allowed to re-entrain to 12/12 h light/dark cycle under bright light (200 lux) for two weeks before going through a novel “jet-lag” paradigm (6-h phase advance) using dim light (10 lux) lasting 14 days. General activity was measured by using infrared motion sensors (Actimetrics, Inc) wired to a computer. Data were collected in 1-min bins using Clocklab software (Actimetrics, Inc).

### Sleep recording

Adult mice (approximately six months old) were chronically implanted with screw-type electrodes in the skull to measure cortical EEG. A pair of stainless-steel electrodes was implanted in the dorsal neck muscle to measure EMG. Screw electrodes were placed in burr holes in the skull over the parietal cortex (–1.5 mm bregma, +1.5 mm midline) and frontal cortex (+1.5 mm bregma, –1.5 mm midline) with a reference electrode over the cerebellum (1.0 mm caudal to λ, 0 mm midline) and a ground over the olfactory bulb area.

Electrodes were connected to head-mounts and secured with dental cement. The animals were allowed to recover from surgery for at least one week, before the EEG/EMG recordings were performed.

At the time of the recordings, mice were tethered to four channel EEG/EMG recording systems (Pinnacle Technology Inc.) and housed individually and sequentially in a soundproof and light-controlled cabinet (standard light conditions 200 lux) equipped with a videocamera with a 3.6-mm lens and infrared illumination (Pinnacle Technology Inc). Data were acquired continuously for a 48-h period, maintaining the same light-dark cycle, temperature and humidity as for the home cages. The EEG/EMG signals were sampled at 250 Hz, amplified 100×, and low-pass filtered at 100 Hz using a two EEG channel, two EMG channel mouse preamplifier (Pinnacle Technology Inc).

Sleep scoring was performed manually on 10-s epochs using Sirenia Sleep software (Pinnacle Technology Inc.). EEG and EMG recordings were synchronized for each epoch to video recordings. Epochs with EMG amplitude slightly (quiet Wake) or significantly higher than baseline (active Wake), together with desynchronized low amplitude EEG were scored as “Wake.” Epochs with low-amplitude EMG and high amplitude δ (1–4 Hz) activity were scored as “NREM” and epochs with low amplitude EMG accompanied by low-amplitude rhythmic θ activity (6–9 Hz) were recorded as “REM” (Quattrocchi et al., 2015).

Distance traveled and velocity were extracted from video files at 30 fps and synchronized with the EEG and EMG data for each individual mouse, using the Sirenia software video plugin (Pinnacle Technology, Inc).

### Electrophysiology on acute brain slices

Animals were culled in accordance with the United Kingdom Home Office Animals (Scientific Procedures) Act 1986 guidelines. Brains were rapidly removed from the skull and immediately immersed in ice cold slicing solution (92 mm NMDG, 2.5 mm KCl, 1.25 mm NaH_2_PO_4_, 30 mm NaHCO3, 20 mm HEPES, 25 mm glucose, 2 mm thiourea, 5 mm Na-ascorbate, 3 mm Na-pyruvate, 0.5 mm CaCl_2_·4H_2_O, and 10 mm MgSO_4_·7H_2_O), pH 7.3–7.4 when bubbled with 95%O_2_/5%CO_2_). Slices were cut using a vibratome tissue slicer (Campden instruments) at a thickness of 300 μm, after which they were immediately transferred to a holding chamber containing slicing NMDG at 33–34°C continuously bubbled with 95%O_2_/5%CO_2_. Slices were left to equilibrate for 10–15 min, after which they were transferred into a holding chamber at room temperature containing recording ACSF (125 mm NaCl, 2.5 mm KCl, 2 mm CaCl_2_, 1 mm MgCl, 1.25 mm NaH_2_PO_4_, 26 mm NaHCO_3_, 11 mm glucose, pH 7.4) that was continuously bubbled with 95%O_2_/5%CO_2_.

Slices were then visualized using a fixed-stage upright microscope (BX51W1, Olympus and Scientifica Slice scope) fitted with a high numerical aperture water- immersion objective and an infra-red sensitive digital camera. A 595-nm amber LED was used for identifying mCherry expression and a 470-nm blue LED was used for optogenetics stimulation. Patch pipettes were made from thick-walled borosilicate glass capillaries (0.86-mm internal diameter, 1.5-mm outer diameter, Harvard Apparatus) using a two-step vertical puller (Narishige, PC-10). Pipette resistances were typically 5–8 MΩ when back filled with internal solution. For voltage-clamp experiments, the internal solution contained: 140 mm CsCl, 4 NaCl mM, 0.5 mm CaCl2, 10 mm HEPES, 5 mm EGTA, 2 Mg-ATP mM; and the pH was adjusted to 7.3 with CsOH. For current-clamp experiments the internal solution contained: 145 mm K-gluconate, 4 mm NaCl, 0.5 mm CaCl_2_, 10 mm HEPES, 5 mm EGTA, 4 mm Mg-ATP, 0.3 mm Na-GTP (adjusted to pH 7.3 with KOH). The amplifier head stage was connected to an Axopatch 700B amplifier (Molecular Devices).

The amplifier current output was filtered at 10 kHz (–3 dB, eight-pole low-pass Bessel) and digitized at 20 kHz using a National Instruments digitization board (NI-DAQmx, PCI-6052E; National Instruments). Data acquisition was performed using CED Signal (version 6) software. CED Signal's “IntraSpikeAnalysis” spike detection script was used for Current Clamp action potential detection thresholding at 0 mV. WinEDR (Strathclyde Electrophysiology Software) was used for Voltage Clamp postsynaptic current detection through template fitting at 0.1 ms (Tau Rise) and 10 ms (Tau Decay).

For peristimulus time histogram (PSTH), an in-house MATLAB code (https://github.com/dd119-ic/BrockManuscript) was used to construct PSTHs from the optogenetic input timings and detected events. OriginPro v2020 was used to construct histograms and for power spectrum analysis.

### Quantification and statistical analysis

#### Monosynaptic viral tracing

Nikon A1R Inverted or Nikon Upright Ni-E confocal optics were used to acquire images using a 20×/NA 0.75 Plan Apo VC or a 60×/1.4 NA objective. Data on starter cells were collected by analyzing z-stack images of all coronal sections spanning the entire injection site, acquired with A1R Nikon confocal microscopes, using the 'multipoint' function in Fiji (Schindelin et al., 2012). Mono-synaptic inputs were calculated as percent of total for each brain or normalized per starter cell, using Excel 365 (Microsoft) and GraphPad Prism 8 software.

The location and distribution of transsynaptically labeled neurons across the brain was assessed using a Zeiss AxioImager microscope using the 4×/0.10 Acroplan and 10×/0.3 Ph1 EC-Plan-NeoFluar objectives. Regions where the GFP^+^ somas were present were identified by comparison with the Paxinos and Franklin *Mouse Brain Atlas*. The Zeiss AxioImager microscope was also used to acquire overviews of coronal sections showing the brain-wide distribution of inputs, using a Plan NeoFluar 2.5×/0.075 objective.

#### RGC analysis

A Nikon A1R confocal microscope was used to acquire z-stacks (step size 1.1 μm) of the RGCs using the 20×/NA 0.75 Plan Apo VC objective. The stacks were acquired such that both ON and OFF ChAT^+^ layers and the entire extent of the RGC's dendrites were included. Overviews of the retinas were acquired using a 10×/NA 0.3 Plan Fluor D objective and images were acquired as z-stacks (step size: 10 μm) and were composed of 4 × 4 tiles.

RGC dendritic arbor tracing and annotation of ChAT layers was based on the protocol published previously (Sumbul et al., 2014). The RGC dendritic trees were traced manually using the Simple Neurite Tracer plugin (Longair et al., 2011; Schindelin et al., 2012) and exported as .swc files. To annotate the ChAT layers, the z-stack images were first resliced so that the z-dimension was projected onto the *y-axis*. The ON ChAT layer was manually annotated, using the 'multipoint' function in Fiji, and every 80th digital slice was marked with 5–10 data points. The x, y and z coordinates for all the points were exported as a .txt file. The procedure was then repeated for the OFF ChAT layer. Opn4 expression levels in RVdG-infected RGCs was compared with the stronger signal from putative M1 ipRGCs and the background signal in the RGC layer, within the same image frame.

RVdG-labeled RGCs were analyzed using a MATLAB implementation of the algorithm developed by (Sumbul et al., 2014) and available at https://github.com/padraic-padraic/rgc. The algorithm begins by “unwarping” the ChAT layers, to produce two flat planes corresponding to the ON and OFF layers. The program then quantifies the arbor density in the IPL relative to these layers, outputting a histogram of arbor density against the IPL *z-axis*, where z = 0μm corresponds to the ON layer and z = 12 μm corresponds to the OFF layer. The stratification data were then processed further, based on Siegert et al. (2009) and Rompani et al. (2017). In particular, the IPL was divided into 10 layers, which were defined such that the OFF ChAT layer is contained in stratum 3, and the ON layer in stratum 7. The remaining layers were defined by linearly interpolating the spacing between the ON and OFF ChAT layers and extending this interpolation to produce 10 full strata corresponding to 3 μm each. The dendritic arbor density histogram was then binned into each of these 10 strata using our custom MATLAB script (https://github.com/padraic-padraic/rgc). Each bin contained the summed arbor density within its range. Stratification above and below the “boxed” region was included in bins 1 and 10, respectively. A stratum was considered “labeled” if the total arbor density in that bin was greater than the average density across all bins. This strata labeling was used to output boxplots of stratification. Cells were then classified into ON, OFF or ON-OFF stratifying using the labeled stratum. ON stratifying cells had only bins 6–10 labeled. OFF stratifying cells had only bins 1–5 labeled. Lastly, ON-OFF stratifying cells had either bins 1–5 and 6–10 or were seen to have clear peaks in ranges corresponding to both ON and OFF stratum bins from raw stratification output.

The diameter of the dendritic arbor was quantified using our custom MATLAB script (https://github.com/padraic-padraic/rgc), based on the skeletonized arbor. The output .swc file contains a description of the arbor as “nodes” connected by “edges.” The ends of the dendritic arbor were identified as all nodes connected to only a single edge. Their x and y co-ordinates were converted to physical distances from the center of the image by multiplying them with the corresponding voxel resolutions in micrometers. Using the MATLAB 'pdist' routine, the Euclidian distance between every pair of end-nodes was calculated, and the maximum value was taken as the dendritic diameter. Scholl analysis and measurements of total branching points and total dendritic length were performed using the SNT plugin for FIJI. Total branching points and total dendritic length were measured using SNTs in-built measurement functions for cable length and number of branch points. In Scholl analysis a pixel central to the nucleus of RGCs was chosen as a starting point and radius step size was set to 10 µm. Statistical comparisons of morphologic parameters from ON, OFF, and ON-OFF stratifying cell groups were made with Kruskal–Wallis tests followed by Dunn's multiple comparisons tests.

#### EEG/EMG data analysis

Cumulative power in α (8–12 Hz), δ (0.5–4 Hz), and θ (6–9 Hz) frequency bands were calculated by computing the discrete Fourier transform (DFT) of the EEG data using a fast Fourier transform (FFT) algorithm. Then, the summed power in each frequency band was normalized to the sum of the power over the entire range (0–15 Hz). Power spectral density (PSD) estimates were calculated using Welch method (window length = 1000; NFFT = 1024). Spectograms showing the amplitude of EEG signals in the time and frequency domain were generated using short-time Fourier transform (window length = 1024; NFFT = 4096), as previously described (Zhivomirov, 2019). θ/δ and δ/θ ratios were calculated by summing PSD values for each frequency range and dividing by each other. EEG signal analyses were conducted using custom codes written in MATLAB, as previously described (Pang et al., 2009; Gelegen et al., 2014, 2018; Zhivomirov, 2019).

#### EEG/EMG statistical analysis

All statistical tests were performed in GraphPad Prism 8. Shapiro–Wilk test was used for normality of distribution. Data are represented as the mean ± SEM, unless otherwise stated. Time spent at each vigilance state, θ/δ and δ/θ ratios for the hour preceding and following the circadian light change was compared between the two groups using paired *t* test or Wilcoxon test, depending on the normality of the data. Normalized power at α, δ, and θ frequency bands for the hour preceding and 2 h following the circadian light change were compared between the groups first using repeated measures one-way ANOVA or Friedman Test depending on the normality of the data. If a significant overall *p* value was obtained, individual time points were compared using paired *t* test or Wilcoxon test.

#### Code availability

The code for RGC analysis generated during this study is available at GitHub https://github.com/padraic-padraic/rgc.

The code for PSTH is available at GitHub https://github.com/dd119-ic/BrockManuscript.

## Results

### The IGL/LGv complex contains cells of thalamic as well as prethalamic origins

**Figure 1.**
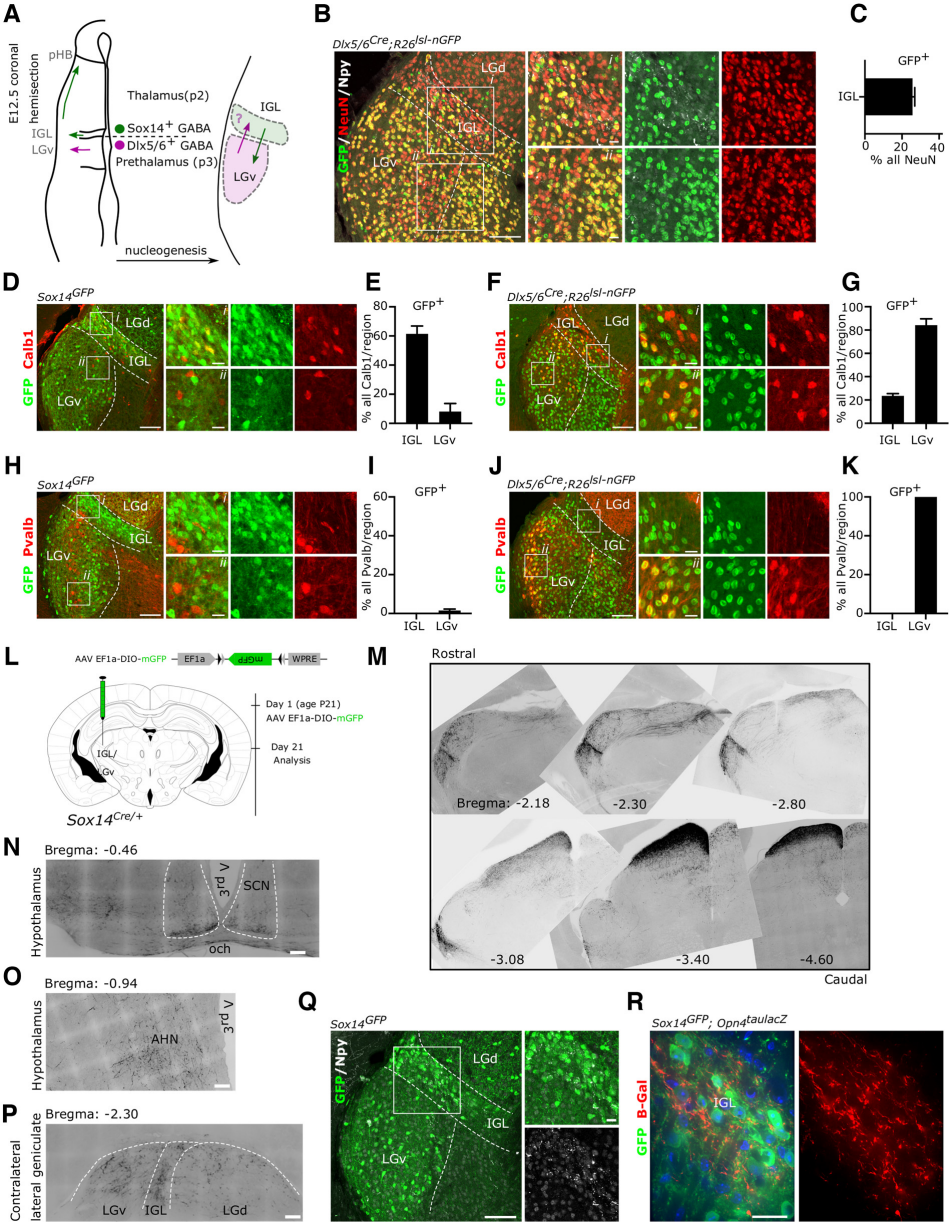
Thalamic and prethalamic lineages in the IGL/LGv complex. ***A***, Schematic representation of the left diencephalon along the coronal plane at the time of peak embryonic neurogenesis (E12.5) illustrating the two main sources of GABAergic neurons for the IGL and the LGv. Radial migration of prethalamic (p3) GABAergic precursors (magenta) generates the bulk of the LGv, while radial migration of thalamic (p2) GABAergic precursors (green) generate the bulk of the IGL. Tangential migration of thalamic GABAergic precursors contributes to the formation of the pHB and to the cellular complexity of the LGv. The possibility of a complementary contribution of prethalamic GABAergic precursors to the IGL is tested using the prethalamic GABAergic driver *Dlx5/6^Cre^*. ***B***, Representative image illustrating the presence of neurons (NeuN^+^) with prethalamic origins (GFP^+^) in the IGL. Note the presence of NeuN^+^GFP^neg^ neurons in the LGv, consistent with the thalamic origin of some LGv neurons and of NeuN^neg^GFP^+^ glia in the LGd. ***C***, Quantification of the fraction of IGL neurons with prethalamic origin. ***D***, ***E***, Representative images and quantification of the mosaic expression of the calbindin protein (Calb1) among thalamic (*Sox14*^*GFP*/+^) lineages in the IGL and LGv. ***F***, ***G***, Representative images and quantification of the mosaic expression of Calb1 in prethalamic (*Dlx5/6^Cre^;R26^lsl-nGFP^*) lineages in the IGL and LGv. ***H*–*K***, Representative images and quantification of the expression of Pvalb in thalamic and prethalamic lineages of the IGL and the LGv. ***L***, Schematic illustration of timeline of the AAV injection strategy used to label axonal projections of the *Sox14*^+^ IGL/LGv neurons in the adult brain. ***M***, Inverted gray scale images from representative rostrocaudal levels of the IGL/LGv, demonstrating the widespread presence of dark GFP labeled fibers (see ***L*** for strategy) projecting away from the IGL/LGv and toward other diencephalic and mesencephalic structures. ***N*–*P***, Higher magnification images from the experiment in ***L*** showing the presence of sparse GFP-labeled fibers in the SCN, the AHN and the contralateral geniculate. ***Q***, an illustrative example of the location of thalamic *Sox14*^+^ neurons in the IGL and the LGv at three weeks of age. ***R***, Example image showing the incoming ipRGC axons (b-gal) in the region occupied by *Sox14***^+^** neurons in the IGL using the *Opn4*^*taulacZ*/+^;*Sox14*^*GFP*/+^ double transgenic mouse.

The IGL and the LGv are thought to arise from distinct progenitor domains in the thalamic prosomere 2 and prethalamic prosomere 3, respectively (Vue et al., 2007; Kataoka and Shimogori, 2008; Virolainen et al., 2012; Puelles et al., 2013). We and others have shown that radial migration of prosomere 2 *Sox14*^+^ precursors into the thalamic mantle zone generates the IGL primordium (Vue et al., 2007; Jeong et al., 2011; Delogu et al., 2012), which includes the neuropetypde Y (Npy)-expressing neuron class (Virolainen et al., 2012). In the developmental window between gestational day (E)11.5 and E14.5, different subsets of *Sox14*^+^ neurons display tangential migratory behavior from this location, reaching the thalamus-epithalamus border first, at E12.5 to coalesce in the presumptive pHB nucleus (Fig. 1*A*) and then, in a subsequent wave of rostroventral migration, seeding the presumptive LGv with neurons of thalamic origin (Vue et al., 2007; Jeong et al., 2011; Delogu et al., 2012; Virolainen et al., 2012; Fig. 1*A*,*Q*). To assess whether prethalamic neurons also contribute to the mature IGL (Fig. 1*A*), we mapped the fate of prethalamic GABAergic lineages in the IGL at postnatal day (P)21, using the prethalamic Cre-driver mouse line *Dlx5/6^Cre^* (Monory et al., 2006; Puelles et al., 2020; Jager et al., 2021) crossed with the conditional reporter line *Rosa26-CAG-Sun1/sfGFP* (*R26^lsl-nGFP^*; Mo et al., 2015). To assist with the anatomic delineation of the IGL, we co-labeled coronal tissue sections of the lateral geniculate with an antibody against Npy and counted the proportion of neurons (NeuN^+^) within IGL boundaries that have prethalamic origin (NeuN^+^nGFP^+^; Fig. 1*B*). This analysis revealed that about a quarter of the neurons in the IGL are of prethalamic origin (Fig. 1*C*; 25.69 ± 1.17%, mean ± SEM, *n* = 3 mice). The calcium binding proteins calbindin (Calb1) and parvalbumin (Pvalb) mark different cell types in the mature IGL/LGv complex (Sabbagh et al., 2020). Using the *Sox14*^*Gfp*/+^ (Delogu et al., 2012) and the *Dlx5/6^Cre^;R26^lsl-nGFP^* reporter lines to label thalamic and prethalamic IGL/LGv lineages, respectively, we noted a similar proportion of Calb1^+^ IGL/LGv neuron subsets in both developmental classes. In the IGL 61.40 ± 5.46% of Calb1^+^ cells belonged to the thalamic *Sox14*^+^ developmental class and 23.53 ± 1.94% to the prethalamic *Dlx5/6*^+^ class; in the LGv 8.1 ± 5.56% belonged to the thalamic *Sox14*^+^ developmental class and 84.17 ± 5.56% to the prethalamic *Dlx5/6*^+^ class (Fig. 1*D–G*; mean ± SEM, *n* = 3 mice per genotype). The Pvalb^+^ subtype was virtually absent from the IGL and found exclusively in LGv lineages of prethalamic origin (Fig. 1*H–K*; *n* = 3 mice per genotype). Hence, while each developmental class clearly differentiates further into several molecularly and functionally distinct cell types (Morin and Blanchard, 1995, 2001; Sabbagh et al., 2020) conventional mature cell markers may not always reflect developmental origin (e.g., Calb1). Importantly, progenitors from the thalamic and prethalamic primordium contribute to the formation of the mature IGL and LGv without clear spatial segregation of developmental lineage classes between the two anatomic regions.

To visualize the pattern of axonal projections from the *Sox14*^+^ IGL/LGv neurons in the mature brain, we injected a Cre-dependent adeno-associated virus (AAV) expressing a cell membrane localised GFP (Matsuda and Cepko, 2007; AAV2/1 Ef1a-DIO-mGFP) in the IGL/LGv of Sox14^*Cre*/+^ mice (Jager et al., 2016) at weaning age and imaged the brain-wide extent of GFP-labeled axons three weeks later (Fig. 1*L*). Although we did not conduct a detailed analysis of axonal projections, we noted that overall, the pattern of efferent projections of the *Sox14*^+^ IGL/LGv neurons was consistent with earlier reports for the anatomically defined IGL and LGv (Moore et al., 2000; Morin and Blanchard, 1995, 1999, 2005). Notably, dense innervation was seen in the superficial gray and optic layers of the superior colliculus (SCs; Fig. 1*M*), while few immunoreactive fibers were present at the ventral edge of the SCN (Fig. 1*N*), sparse and diffuse in the hypothalamus (e.g., anterior hypothalamic nucleus, AHN; Fig. 1*O*) and in all three subdivisions of the contralateral lateral geniculate (LGN; Fig. 1*P*).

We had previously shown that *Sox14*^+^ neurons in the IGL/LGv establish synaptic connectivity within the nonimage-forming circuitry that mediates the pupillary light reflex (Delogu et al., 2012). Taking advantage of the strong GFP expression from the *Sox14*^*Gfp*/+^ mouse reporter line in the juvenile brain (Fig. 1*Q*), we crossed this reporter line with the *Opn4*^*taulacZ*/+^, which labels mostly the M1 subtype of ipRGCs (Hattar et al., 2002, 2006; Baver et al., 2008). The *Opn4*^*taulacZ*/+^;*Sox14*^*Gfp*/+^ double transgenic mouse line confirmed the presence of GFP^+^ neurons within axonal projections labeled by the *taulacZ* reporter construct (Fig. 1*R*), consistent with several reports that propose M1 ipRGC innervation of the IGL/LGv. However, the discovery of additional prethalamic lineages in the IGL (Fig. 1*B*,*C*) raises the possibility that developmentally defined cell classes may receive selective ipRGC-subtype innervation. The *Sox14Gfp* and *Sox14Cre* mouse lines are suitable tools to resolve the developmental complexity of the IGL/LGv by restricting genetic labeling and manipulations exclusively to neurons of thalamic origin.

### Retinal input to the *Sox14*^+^ IGL/LGv originates from non-M1 ipRGCs

**Figure 2.**
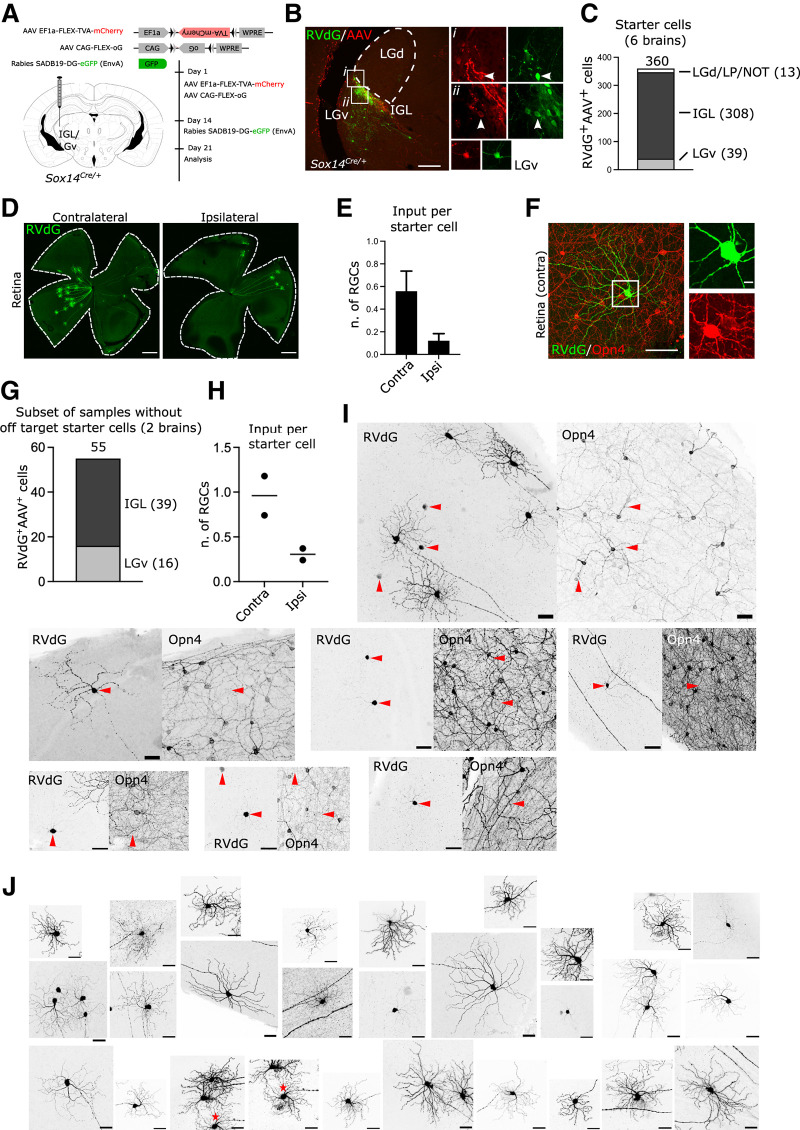
Transsynaptic labeling of retinal input to *Sox14*^+^ neurons in the IGL/LGv. ***A***, Scheme of rabies tracing of IGL/LGv input in *Sox14*^*Cre*/+^ line, showing the location and timeline of the injections and viral vectors used. ***B***, Representative coronal section of virally encoded fluorophores at the injection site, showing RVdG (GFP, green) and helper AAVs (mCherry, red) double-positive neurons within the IGL (white arrowheads) and LGv. Scale bar: 100 µm. ***C***, Total count of primary infected cells in the target and off-target regions from serial confocal images of all sections containing thalamic tissue (*n* = 6 mice). ***D***, Representative images of whole mount retinas showing the presence of RVdG-infected RGCs in the retinas ipsilateral and contralateral to the injected IGL/LGv. Scale bars: 500 µm. ***E***, Number of labeled RGCs per starter cell in the ipsilateral and contralateral retinas (*n* = 6 retina pairs). ***F***, Example image of a retrogradely labeled RGC (green) with weak expression of melanopsin (Opn4, red) in its soma. Note the presence in the same field of view of other RGCs with much higher levels of melanopsin expression. Scale bars: overview image 100 µm, inset 10 µm. ***G***, Total count and distribution of primary infected cells from serial confocal images of all sections containing thalamic tissue in two samples with no detectable off target starter cells (*n* = 2 mice). ***H***, Number of labeled RGCs per starter cell in the ipsilateral and contralateral retinas (*n* = 2 retina pairs). ***I***, Examples of RVdG infected RGCs (RVdG) and the location within the same field of view of ipRGCs with high melanopsin expression (Opn4). Red arrowheads indicate RVdG-infected RGCs for which signal was too weak to allow for morphologic characterization of the dendritic tree, however, in all such cases no strong Opn4 signal was detected. Scale bars: 50 µm. ***J***, Examples of dendritic morphologies of RVdG-infected cells. * denotes the same cell in two frames. Scale bars: 50 µm.

We sought to investigate the extent and diversity of retinal input to the thalamic component of the IGL/LGv, defined by *Sox14* expression. For this purpose, we applied the modified rabies virus technology (Fig. 2*A*), guiding primary infection of a glycoprotein-deleted, GFP-expressing and avian-pseudotyped rabies (SADB19 ΔG-eGFP, EnvA; in short RVdG) to the *Sox14* neurons of the IGL/LGv. Target neurons were primed for RVdG infection by stereotaxic injection in *Sox14*^*Cre*/+^ transgenic mice of equimolar amounts of two Cre-dependent AAVs (Fig. 2*A*) expressing the avian receptor TVA linked via a self-cleavage peptide sequence to the red fluorescent reporter mCherry (AAV2/1 Ef1a-flex-TVA-mCherry) and the codon-optimized version of the rabies glycoprotein (G) gene (AAV2/1 CAG-flex-oG). Our injection strategy reliably targeted the IGL/LGv, as indicated by the cumulative total number of primary infected neurons (GFP^+^mCherry^+^; Fig. 2*B*) from all thalamus-containing sections (IGL: 308, LGv: 39; *n* = 6 brains; Fig. 2*C*), while off target labeling was occasionally observed in some of the sparse *Sox14*^+^ thalamic interneurons along the trajectory of the stereotaxic injection [Fig. 2*C*; LGd, LP, nucleus of the optic tract (NOT): 13; *n* = 6 mice].

While it is known that M1 and non-M1 classes of melanopsin-type ipRGCs project to the IGL/LGv (Hattar et al., 2006; Brown et al., 2010; Ecker et al., 2010; Stabio et al., 2018; Quattrochi et al., 2019; Beier et al., 2020), it is unclear whether RGC subtype input to the IGL/LGv neurons correlates with the developmental origins of IGL/LGv cells. We therefore set out to investigate the transsynaptic spread of the RVdG to the retina and noted consistent labeling of these cells in the contralateral eye and to a lesser extent in the ipsilateral eye (Fig. 2*D*). Normalized RGC input was 0.56 ± 0.18 in the contralateral eye and 0.12 ± 0.06 in the ipsilateral eye (Fig. 2*E*; input per starter cell; mean ± SEM, *n* = 6 mice).

Next, we screened RVdG-infected RGCs for melanopsin expression by immunohistochemical detection of melanopsin on whole mount retinas and noted that none had the high levels of melanopsin expression typically associated with the M1 class; furthermore, dendritic morphologies did not resemble the stereotypical organization characteristic of the M1 class (Fig. 2*F*; 261 cells, 12 retinas; *n* = 6 mice). However, moderate to weak expression was often, but not always, observed in RVdG-labeled RGCs (Fig. 2*F*).

The absence of any obvious M1 input to the *Sox14*^+^ IGL/LGv was unexpected, as *Opn4*^*taulacZ*/+^;*Sox14*^*Gfp*/+^ mice show that *Sox14*^+^ neurons and axons from mostly M1 ipRGCs are clearly present in that same area of the geniculate (Fig. 1*M*). Furthermore, numerous reports have hypothesized M1 specific innervation of the IGL/LGv (Hattar et al., 2006; Ecker et al., 2010; Chen et al., 2011).

There exists the possibility that our transsynaptic labeling of the RGCs is heavily skewed toward the small number of off target primary infected neurons detected in the LGd, LP, and NOT (Fig. 2*C*), which could explain the absence of M1 input in favor of other melanopsin and nonmelanopsin RGC subtypes known to project to thalamic visual areas. However, this seems an unlikely eventuality, because restricting the analysis to two out of the total six brains that had no detectable off target starter cells (Fig. 2*G*,*H*), confirmed that transsynaptic spread to the retina did not involve RGCs with high melanopsin expression or M1 morphology (Fig. 2*I*,*J*).

**Figure 3.**
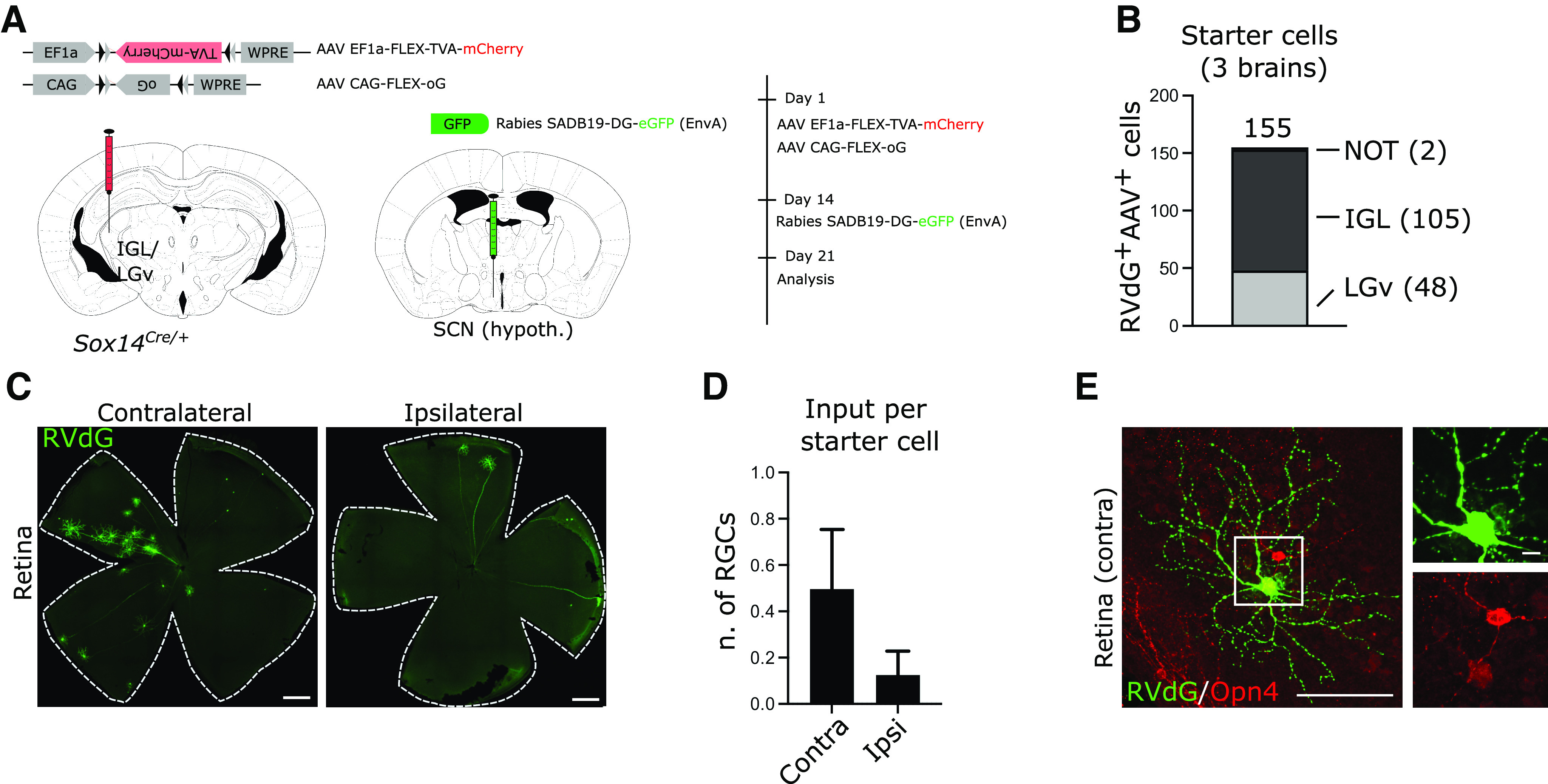
***A***, Scheme of rabies tracing of the input to the IGL/LGv subset with hypothalamic projections, showing the location and timeline of viral injections. ***B***, Total count of primary infected cells in the target and off-target regions from serial confocal images of all sections containing thalamic tissue (*n* = 3 mice). ***C***, Representative images of whole mount retinas showing the presence of RVdG-infected RGCs in the retinas ipsilateral and contralateral to the injected IGL/LGv on RVdG injection in the SCN region. Scale bars: 500 µm. ***D***, Number of labeled RGCs per starter cell in the ipsilateral and contralateral retinas after the injection strategy in ***A*** (*n* = 3 retina pairs). ***E***, Example image of a retrogradely labeled RGC (green) with weak expression of melanopsin (Opn4, red) in its soma, after RVdG injection in the SCN region. Scale bars: overview image 100 µm, inset 10 µm.

We addressed further the possibility of off target starter cells that may have been present but undetectable for technical reasons, by modifying the RVdG labeling strategy to deliver the Cre-dependent AAVs in the IGL/LGv, but the RVdG in the ipsilateral SCN (Fig. 3*A*). While this viral delivery strategy did not result in the exclusive targeting of the SCN, the spread of the injected RVdG viral solution affected only the hypothalamic territory adjacent to the SCN. Hence, by injecting the RVdG in a well-known and distant target of the IGL/LGv we ruled out the possibility of detecting retinal input to the sparse *Sox14*^+^ interneurons of thalamocortical nuclei. This modified injection strategy reliably labeled hypothalamus-projecting *Sox14*^+^ IGL/LGv, as indicated by the cumulative total number of primary infected neurons from all thalamus-containing sections (three mice) in the target region (Fig. 3*B*; IGL: 105, LGv: 48), with residual off target labeling observed in *Sox14*^+^ neurons in the NOT region (Fig. 3*B*; two cells). RVdG infected RGCs were detected in all brains analyzed (Fig. 3*C*), with the following normalized distribution: contralateral RGCs 0.50 ± 0.26 and ipsilateral RGCs 0.12 ± 0.1 (Fig. 3*D*; mean ± SEM, *n* = 3 mice). Inspection of the retinas did not reveal any obvious M1 dendritic morphology, nor was strong melanopsin expression seen in any of the RVdG-infected RGCs (Fig. 3*E*; 39 cells, *n* = 3 mice).

**Figure 4.**
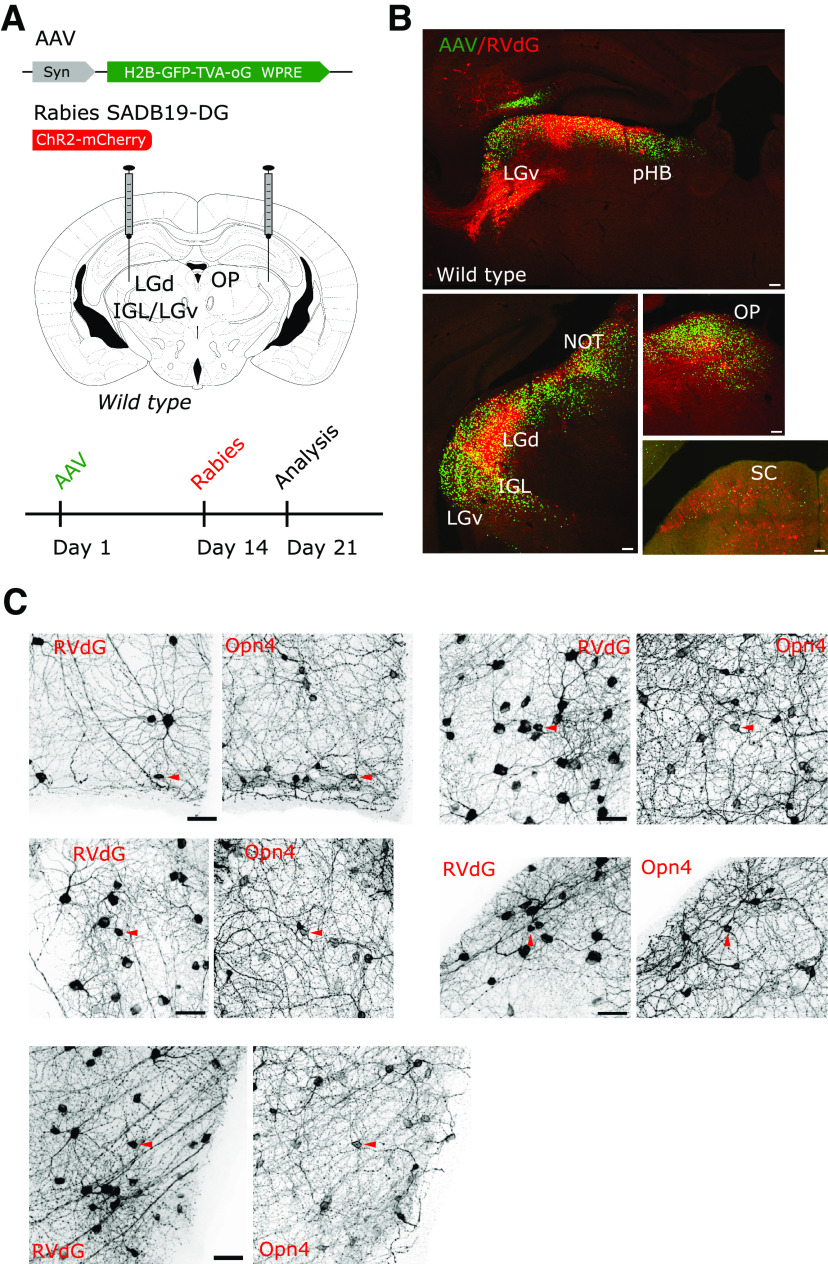
Retrograde transsynaptic labeling of the retina independent of the genetic identity of the starter cells. ***A***, Scheme of the delivery strategy of a constitutive AAV and monosynaptic restricted rabies to the retinorecipient regions of the prethalamus, thalamus, pretectum, and midbrain, showing the timeline of viral injections. ***B***, Infection with the constitutively active helper AAV can be detected by its expression of nuclear-restricted GFP (green) in several retinorecipient areas of the diencephalon and midbrain. Primary infection with a RVdG-mCherry2 virus (red) is detected abundantly in the retinorecipient LGd, IGL, pHB, OP and to a lesser extent in the LGv and SC. Scale bars: 100 µm. ***C***, Transsynaptic spread of the RVdG to the retinas labels large numbers and types of RGCs, including those with high melanopsin (Opn4) expression (arrowheads). Images are a representative example from one tracing experiment out of three with similar infection patterns. Scale bars: 50 µm.

The notable absence of M1 ipRGCs from the pool of RVdG-labeled cells may reflect a yet unreported negative tropism of the SADB19 rabies strain, rather than specific subtype connectivity. However, this possibility seems unlikely, because virally infected M1 ipRGCs have been detected after injection of the RVdG in the thalamus of mice (Fernandez et al., 2018) and macaques (Dhande et al., 2019). Furthermore, we confirmed that the RVdG can infect M1 type ipRGCs by performing injection of a non-Cre-dependent helper AAV (AAV2/1 Syn-H2B-GFP-TVA-oG-WPRE3) in wild-type Bl6C57 mice (*n* = 3) to target a large volume of the diencephalon, followed by injection of RVdG (RVdG-ChR2-Cherry) in the same region of the brain (Fig. 4*A*,*B*). Such tracing strategy of the retinal radiation led to widespread labeling of RGCs that included bona fide M1 ipRGCs with high Opn4 expression (Fig. 4*C*). This last observation therefore reinforced our conclusion that the thalamic component of the IGL/LGv, including neurons that project to the SCN area of the hypothalamus, lacks an obvious M1 input and implies that this important source of luminance information may depend on a different and parallel *Sox14^neg^* IGL/LGv circuitry.

**Figure 5.**
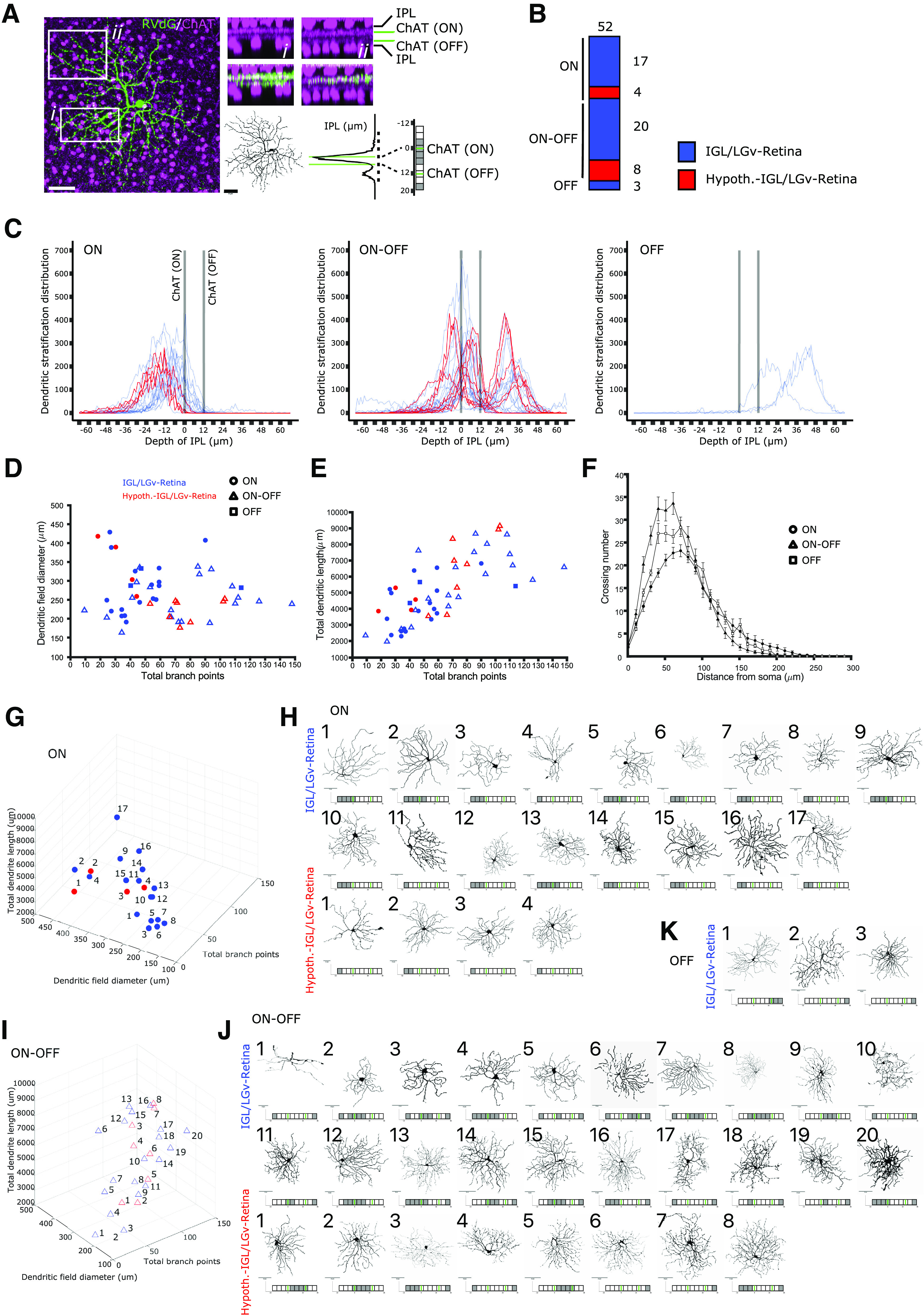
Dendritic morphology of RGCs projecting to *Sox14*^+^ neurons in the IGL/LGv. ***A***, Example images illustrating the strategy for the morphologic analysis of RGC dendrites and their stratification in the IPL. The IPL is visualized sandwiched between ChAT^+^ amacrine cell somas (magenta) for two arbitrary regions (*i* and *ii*) of an RVdG infected RGC (green). The dendritic reconstruction of the RGC is presented as black skeleton and the raw density of the GFP signal in the IPL is subdivided along 10 bins using gray color for thresholding. Green lines represent ChAT^+^ ON and OFF laminar references. Scale bars: 50 µm. ***B***, Morphologic analysis was conducted for RGCs that did not have overlapping dendrites with other labeled RGCs. Graphical representation of the proportion of ON, ON-OFF and OFF stratifying RGCs, color coded in blue when labeling occurred after RVdG injection in the IGL/LGv and in red when labeling occurred after RVdG injection in the hypothalamic region of the SCN (cumulative values from 9 mice). ***C***, Distribution of dendritic densities for each of the 52 reconstructed RGCs, grouped according to their stratification in the ON and OFF laminas of the IPL and color coded in blue or red as in ***B***. ***D***, ***E***, Scatter plots displaying the dendritic field diameter and total dendritic length against the total dendritic branch points for all 52 reconstructed RGCs, color coded in blue or red as in ***B***. Filled circles represent the cells in the ON cluster, triangles the cells in the ON-OFF cluster and squares the cells in the OFF cluster. ***F***, Scholl analysis for the three clusters of RGCs, without color coding to indicate location of the RVdG injection. ***G***, 3D rendering of the morphologic features in ***D***, ***E*** for the ON RGC cluster, color coded in blue or red according to the tracing strategy. ***H***, Reconstructed dendritic trees and dendritic stratification profiles of retrogradely traced RGCs in the ON cluster, numbered according to their increasing branch points and divided according to the labeling strategy. Scale bars: 50 µm. ***I***, 3D rendering of the morphologic features in ***D***, ***E*** for the ON-OFF RGC cluster, color coded in blue or red according to the tracing strategy. ***J***, Reconstructed dendritic trees and dendritic stratification profiles of retrogradely traced RGCs in the ON-OFF cluster, numbered according to their increasing branch points and divided according to the labeling strategy. Scale bars: 50 µm. ***K***, Reconstructed dendritic trees and dendritic stratification profiles of retrogradely traced RGCs in the OFF cluster which is only labeled by the RVdG injection in the IGL/LGv. Scale bars: 50 µm.

To further describe the morphologic features of RVdG-labeled RGCs, we quantified radial dendritic morphology and dendritic stratification in the inner plexiform layer (IPL) relative to the ChAT^+^ ON layer and the ChAT^+^ OFF layer of amacrine cell processes (Sumbul et al., 2014; Fig. 5*A*). The stratification of the IPL was divided in 10 layers (Siegert et al., 2009; Rompani et al., 2017) so that ChAT^+^ ON and OFF layers match strata 7 and 3, respectively. A custom MATLAB script (kindly provided by Padraic Calpin, UCL) was used to bin the summed arbor density into each of these 10 strata and thresholding applied to color-code densely populated bins (Fig. 5*A*; gray). We selected 40 RGCs from animals with AAVs and RVdG injected in the IGL/LGv (six mice) and 12 RGCs from animals with AAVs injected in the IGL/LGv and RVdG injected in the hypothalamic SCN region (Fig. 5*B*; *n* = 3 mice). Criterium for selection of the RGCs was their spatial segregation from other labeled RGCs, so that dendritic morphologies could be reliably reconstructed. We then grouped all reconstructed RGCs into three classes based on whether their dendritic stratification aligned with the ChAT ON lamina (ON), both the ChAT ON and OFF (ON-OFF) or the ChAT OFF lamina (OFF; Fig. 5*B*,*C*); hence, the nomenclature adopted is not intended to reflect physiological properties, but dendritic stratification only. Plotting of the dendritic distribution in the IPL for the three groups clearly supports our initial observation of lack of M1 type input, as only three cells belonged to the OFF class, none of which was labeled by the hypothalamic injection of the RVdG (Fig. 5*C*). The ON group contained 21 RGCs, 17 labeled by injecting the RVdG in the IGL/LGv and four labeled by injecting it in the SCN hypothalamic region (Fig. 3*B*,*C*). The ON-OFF group contained 28 RGCs, of which 8 were labeled by the SCN hypothalamic injection of the RVdG (Fig. 5*B*,*C*). While, the ON class displayed more homogenous dendritic stratification, the ON-OFF class appears clearly heterogeneous, with bistratified RGCs as well as RGCs showing broad dendritic density spanning the ChAT ON and OFF layers (Fig. 5*C*). To further characterize the types of RGCs in each of the three groups, we measured the diameter of the dendritic field, the total length of the dendritic tree and the number of dendritic branchpoints (Fig. 5*D*,*E*). We then performed Scholl analysis to measure dendritic complexity at increasing distances from the cell soma (Fig. 5*F*). We did not measure soma size, because of the heavily saturated GFP signal of the cell somas in our confocal images. RGCs in the ON group have dendritic features consistent with ON stratifying M2, M4, and M5 ipRGCs (Fig. 5*D*,*E*,*G*; range of branchpoints: 18–90, range of dendritic tree diameter: 191.2–428.5, range of total dendritic length: 2301–6821) in line with other reports for these ipRGC subtypes (Berson et al., 2010; Ecker et al., 2010; Schmidt and Kofuji, 2011; Estevez et al., 2012; Stabio et al., 2018). Overall, RGCs in the ON-OFF group displayed broader morphologic heterogeneity and significantly larger total dendritic length and branch points compared with the ON group (branch points: *p* = 0.0005; total dendritic length: *p* = 0.012; field diameter: *p* = 0.031; Kruskal–Wallis test). Stratification of the dendritic tree in the IPL discriminates M3 ipRGCs from similarly complex M2 dendrites (Schmidt and Kofuji, 2011). While some of the RGCs in the ON-OFF group display parameters compatible with the M3 type, other RGCs display higher branch points and dendritic length compatible with the recently described M6 ipRGCs (Quattrochi et al., 2019). We cannot exclude that non-ipRGCs are also present in this group, for instance subsets of ON-OFF direction selective (DS) RGCs. The LGv is one of the targets of ON-OFF DS-RGCs (Huberman et al., 2009; Rivlin-Etzion et al., 2011; Dhande et al., 2019) that could potentially mediate some of the nonimage forming functions of this RGC class (Rivlin-Etzion et al., 2011).

In summary, reconstruction and quantitative analysis of dendritic morphologies of isolated RGCs retrogradely labeled from *Sox14*^+^ IGL/LGv neurons shows that OFF stratifying M1 ipRGCs are not an obvious source of luminance information (Fig. 5*C*,*K*), while heterogenous retinal input is mostly from ON (Fig. 5*C*,*G*,*H*) and ON-OFF (Fig. 5*C*,*I*,*J*) stratifying RGCs. Several of the RGCs analyzed here have morphologic features compatible with non-M1 types of ipRGCs. However, we cannot exclude that other RGCs are also a source of retinal input to the *Sox14*^+^ IGL/LGv.

### Brain-wide input to the *Sox14*^+^ IGL/LGv is skewed toward visual networks

**Figure 6.**
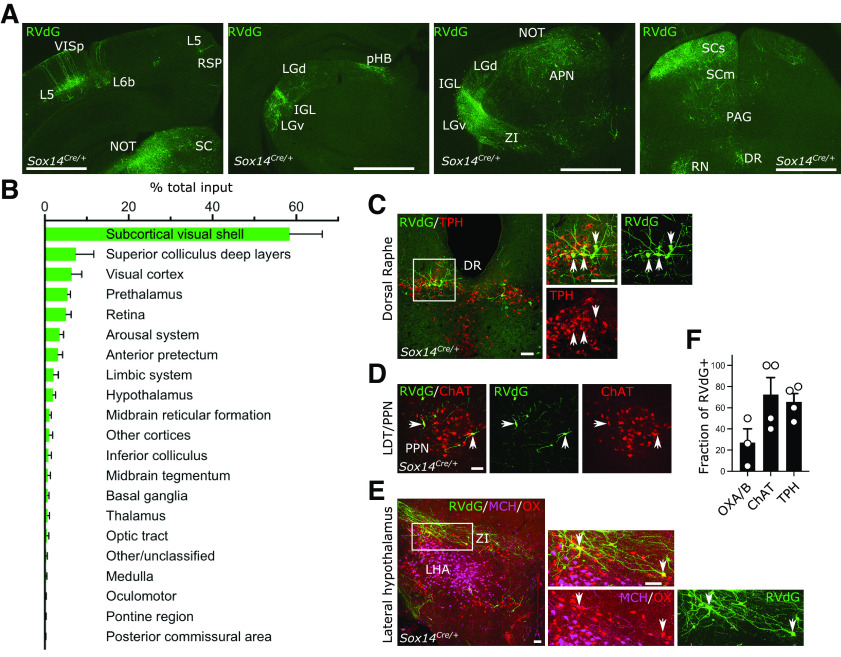
Brain-wide input to the *Sox14*^+^ neurons in the IGL/LGv. ***A***, Representative coronal sections showing brain-wide distribution of inputs to *Sox14*^+^ neurons in IGL/LGv. Most inputs to *Sox14*^+^ IGL/LGv can be observed in areas with visual functions (VISp, SCs, NOT, IGL/LGv). Green, GFP from RVdG. Scale bars: ∼1 mm. ***B***, Quantification of inputs to *Sox14*^+^ neurons in IGL/LGv, shown as percentage of total inputs (mean ± SEM; *n* = 4 mice). See also Extended Data Table 6-1 for anatomic classification based on previously published work (Paxinos and Franklin, 2001). ***C***, ***D***, Representative coronal sections showing inputs from the ascending arousal system. Not all RVdG-infected cells (GFP^+^, green) are co-stained with the anti-tryptophan hydroxylase (TPH; red) antibody in dorsal raphe (DR) or the anti-choline acetyltransferase (ChAT; red) in pedunculopontine nucleus (PPN). Scale bars: 100 mm. ***E***, Representative coronal section showing inputs from the lateral hypothalamus. In the lateral hypothalamic area, a small proportion of RVdG-infected cells (GFP^+^, green) are co-stained with the anti-orexinA/B antibody (OX, red), but none with the anti-melanin concentrating hormone antibody (MCH, magenta). Scale bars: 100 µm. ***F***, Quantification of the fraction of RVdG-labeled neurons that expressed the indicated markers (mean ± SEM).

The IGL/LGv is thought to receive and integrate photic information from the retina with information pertaining to the internal state of an organism, which ascends via the brainstem arousal system. Hence, we systematically analyzed the range and proportion of afferents to the *Sox14*^+^ neurons of the IGL/LGv by mapping the location of all transsynaptic RVdG-infected cells (GFP^+^) in 4 of the 6 mice used for tracing of the retinal input (Fig. 2*A*). The vast majority of the afferents arise ipsilaterally in the diencephalic compartments: in the hypothalamus, the zona incerta (ZI) and LGv in the prethalamus, the IGL (including contralateral) and the pHB in the thalamus, the anterior pretectal nucleus (APN) and the NOT in the pretectum (Fig. 6*A*,*B*). More than half of the inputs can be grouped under a grossly visual functional classification that includes the retinorecipient subcortical visual shell (Extended Data Table 6-1; including the superficial layers of the superior colliculus (SCs), the IGL/LGv, the olivary pretectal nucleus (ON) and the NOT, the cortical pyramidal neurons in layer 5 (L5) and L6b of the primary visual area (VISp; Fig. 6*A*,*B*) and direct retinal input (Fig. 2*D*).

10.1523/JNEUROSCI.0112-21.2022.tab6-1 Extended Data Table 6-1 Anatomical classification of regions harboring presynaptic input to *Sox14*^+^ neurons. The classification used to cluster anatomical regions containing cells transsynaptically labeled by the RVdG vector is based on the atlas of the mouse brain by Paxinos and Franklin (Paxinos and Franklin, 2001). Download Table 6-1, DOCX file.

In contrast, input from the ascending arousal system only accounted for <5% of the total (Fig. 6*B–E*; Extended Data Table 6-1). Closer inspection revealed this input originates mostly in the serotonergic tryptophan hydroxylase (TPH)^+^ dorsal Raphe (DR; Fig. 6*C*) and the cholinergic (ChAT^+^) pedunculopontine nucleus (PPN; Fig. 6*D*). Although input from the locus coeruleus to the IGL has been proposed (Morin, 2013), we could not reliably detect it for the *Sox14*^+^ subtype. Furthermore, we noted that not all the RVdG infected neurons in the PPN expressed the cholinergic marker ChAT (Fig. 6*F*; 72.5 ± 16.01%, mean ± SEM). Of the input arising in the DR, 65.7 ± 7.68% GFP^+^ cells co-expressed the marker TPH (Fig. 6*F*; mean ± SEM). In the lateral hypothalamic area (LHA), a small fraction of the retrogradely labeled input was from orexinergic (OX) neurons (Fig. 6*F*; 27.06 ± 13.0%, mean ± SEM) and none was from melanin concentrating hormone (MCH) neurons (Fig. 6*E*), in agreement to an earlier report in hamsters (Vidal et al., 2005). In summary, the monosynaptic input to *Sox14*^+^ neurons in the IGL/LGv originates overwhelmingly from vision-related structures and to a limited extent from the brainstem's ascending arousal and neuromodulatory systems.

### The *Sox14*^+^ IGL/LGv is required for circadian re-entrainment in presence of weak photic cues

In mammals, alignment of circadian physiology and behavior with the daily light cycle depends on rod, cones and melanopsin retinal photoreceptors and on specific ipRGC to brain connectivity so that free running circadian rhythms are observed only when all photoreceptors are simultaneously inactivated or ipRGCs selectively ablated (Panda et al., 2003; Güler et al., 2008; Hatori et al., 2008). Melanopsin loss of function mutations alone are not sufficient to cause an overt photoentrainment phenotype, but result in reduced pupillary constriction (Lucas et al., 2003) and reduced behavioral responses to acute light exposure (Panda et al., 2002; Mrosovsky and Hattar, 2003; Altimus et al., 2008; Lupi et al., 2008). The thalamic contribution to circadian photoentrainment is not fully elucidated. Our data showing the notable lack of M1 ipRGC input to the *Sox14*^+^ neurons of the IGL/LGv and limited innervation from the ascending arousal system represent unexpected findings that pose the question of the extent to which this developmentally defined subset of the IGL/LGv complex can contribute to circadian entrainment of motor activity rhythms.

**Figure 7.**
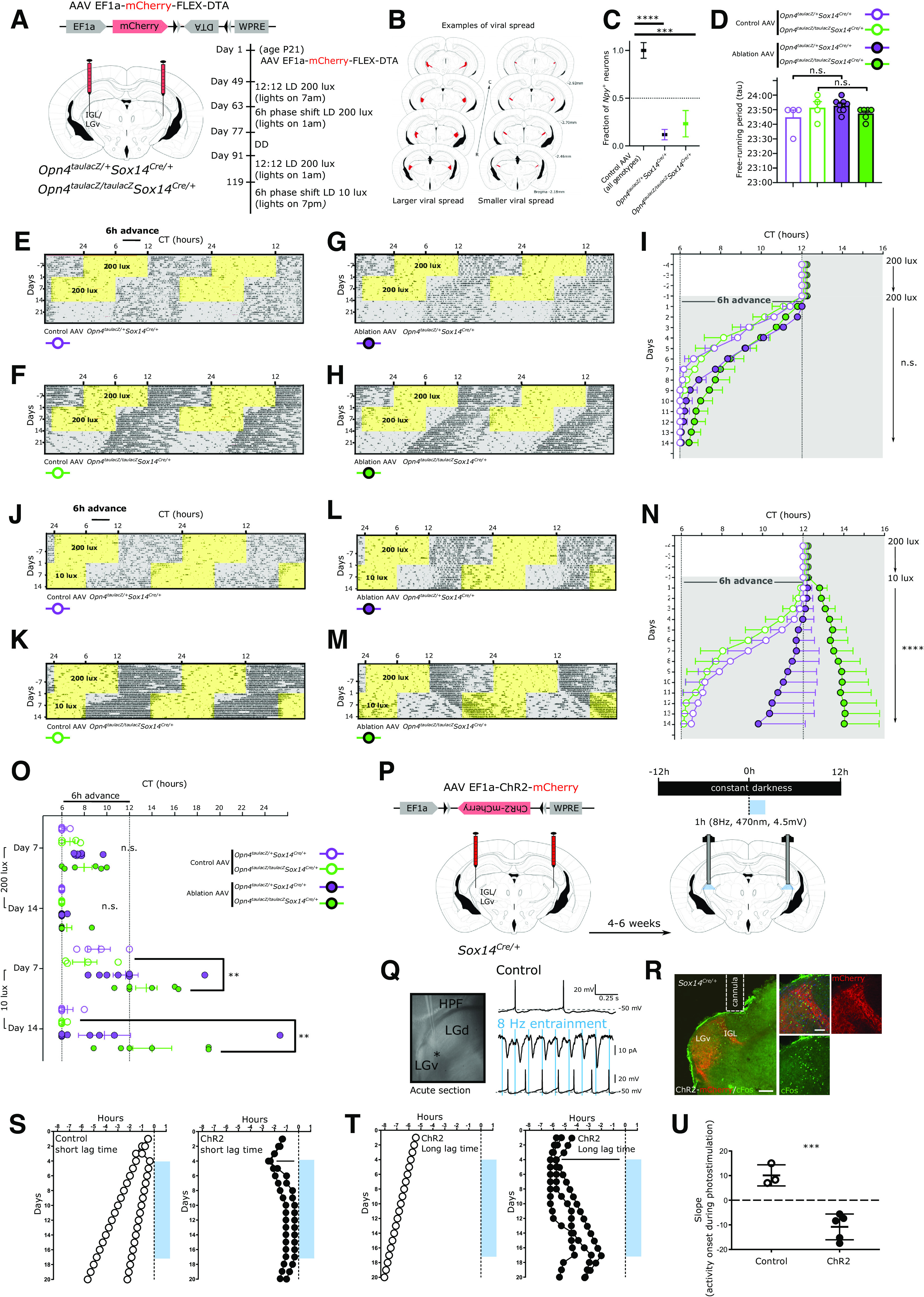
Perturbation of the *Sox14*^+^ IGL/LGv neurons leads to aberrant onset of circadian motor activity rhythms. ***A***, Scheme for the specific ablation of *Sox14*^+^ IGL/LGv neurons in the melanopsin loss of function and control background and timeline of the circadian light paradigms. ***B***, Evaluation of the viral spread on stereotaxic injection in the IGL/LGv region, in two representative brains depicting the extent of mCherry expression (red), which is not Cre-dependent, in a case of more extensive viral infection (left) and one of less extensive infection (right). ***C***, Quantification of the residual fraction of IGL *Npy*^+^ cells, setting the mean value for nonablated samples at 1. Controls combined: *Opn4*^*taulacZ*/+^*Sox14*-control and *Opn4^taulacZ/taulacZ^Sox14*-control *n* = 8 mice, *Opn4*^*taulacZ*/+^*Sox14*-ablated *n* = 7 mice, *Opn4^taulacZ/taulacZ^Sox14-*ablated *n* = 5 mice. One-way ANOVA *F*_(32.53)_; *p* < 0.0001; mean ± SEM ****p < 0.0001; ***p = 0.0003; *t* test. ***D***, Similar period length under constant dark conditions for all animals in the four groups (n.s.; ANOVA). ***E–H***, Actograms for a representative mouse per group illustrating the activity rhythms under 200 lux, after a 6-h phase advance and in constant darkness. Period of lights on are in yellow, periods of lights off are in gray. ***I***, Onset of circadian motor activity for the four groups, color coded as in ***D***. Periods of lights off are in gray. Days 1–14: n.s.; ANOVA. Data plotted as mean ± SEM. ***J–M***, Actograms for the same representative mice as in ***E–H*** illustrating the activity rhythms under 200 lux, after a 6-h phase advance with concomitant reduction of ambient luminance to 10 lux. ***N***, Onset of circadian motor activity for the four groups. Periods of lights off are in gray. Days 1–14: *p* < 0.0001; Kruskal–Wallis test. *Opn4^taulacZ/taulacZ^Sox14-*ablated significantly different in all pairwise comparisons. Data plotted as mean ± SEM. ***O***, Onset of circadian activity rhythms for each animal in the four groups at day 7 and day 14 after the 6-h phase advance in the light cycle either in standard luminance (200 lux) or dim luminance (10 lux). At 200 lux, a trend toward delayed circadian onset of activity was detected at day 7; however, this was not statistically significant (*p* = 0.041; Kruskal–Wallis test; n.s. after multiple comparison correction). At 10 lux, a high degree of interindividual variability was observed at day 7 and day 14 in the onset of circadian activity rhythms for both the *Opn4*^*taulacZ*/+^*Sox14-*ablated and the *Opn4^taulacZ/taulacZ^Sox14-*ablated groups, which included cases of period lengthening and irregular patterns of activity onset (day 7: *F*_(4.294)_, *p* = 0.019, ANOVA; Tukey's multiple comparisons test *p* = 0.021 for the *Opn4^taulacZ/taulacZ^Sox14-*ablated group; day 14: *p* = 0.0082 Kruskal–Wallis test; Dunn's multiple comparisons test *p* = 0.021 for the *Opn4^taulacZ/taulacZ^Sox14-*ablated group vs *Opn4^taulacZ/taulacZ^Sox14-*control group and *p* = 0.028 for the *Opn4^taulacZ/taulacZ^Sox14-*ablated group vs *Opn4*^*taulacZ*/+^*Sox14-*control group). Data plotted as mean ± SEM. ***P***, Schematic strategy for the expression of ChR2 in *Sox14*^+^ IGL/LGv neurons and the circadian optogenetic stimulation (blue line, 1 h/d, 470 nm, 8 Hz). ***Q***, Bright-field image of the acute slice preparation indicating the location (asterisk) of whole-cell recordings from mCherry expressing neurons within the IGL/LGv. The top voltage trace was obtained from a single neuron in the IGL/LGv showing spontaneous AP generation in the absence of blue light stimuli. The middle trace shows the ChR2-mediated currents elicited by blue light stimulation and the bottom voltage trace demonstrates the resulting entrainment of APs at 8-Hz optogenetic stimulation. ***R***, Representative images from an injected *Sox14*^*Cre*/+^ mouse showing the location of virally infected cells (mCherry, red) in the IGL/LGv region and optogenetically induced c-Fos expression (green), 90 min after the end of the stimulation. Scale bars: 100 µm. ***S***, Daily onset of circadian activity in two control subjects (white circle, left graph) and two subjects expressing ChR2 within *Sox14*^+^ IGL/LGv neurons (black circles, right panel), housed under constant dark conditions. Onset of optogenetic stimulation occurred within the first 3 h of the subjective night. ***T***, Daily onset of circadian activity in one control subject (white circle, left graph) and three subjects expressing ChR2 within *Sox14*^+^ IGL/LGv neurons (black circles, right panel), housed under constant dark conditions. Onset of optogenetic stimulation occurred ∼6 h into the subjective night. ***U***, Plot showing the slope of activity onset over 14 d of optogenetic stimulation in control and ChR2-expressing mice. Positive values indicate negative drifting typical of free running rhythms under constant darkness, whereas negative values reflect a switch to positive drifting. Control group: 10.11 ± 2.48, ChR2-expressing group: −11.38 ± 1.58, *p* = 0.0002; mean ± SEM; *t* test.

We aimed to test the requirement of the IGL/LGv *Sox14*^+^ neurons in circadian photoentrainment under normal laboratory lighting conditions (200 lux) and under reduced luminance (10 lux) or photodetection (melanopsin loss of function). To achieve this, we crossed the *Opn4^taulacZ/taulacZ^* and *Opn4*^*taulacZ*/+^;*Sox14*^*Cre*/+^ mouse lines to generate a *Opn4*^*taulacZ*/+^;*Sox14*^*Cre*/+^ cohort with functional melanopsin expression and a *Opn4^taulacZ/taulacZ^*;*Sox14*^*Cre*/+^ cohort lacking melanopsin expression. We then induced selective apoptosis of *Sox14*^+^ IGL/LGv neurons by injecting bilaterally in the IGL/LGv region an AAV that expresses the diphtheria toxin A subunit in a Cre-dependent manner and the fluorescent reporter mCherry constitutively (AAV2/1 Ef1a-mCherry-DIO-DTA; Fig. 7*A*). Control animals from both cohorts were injected with a Cre-dependent AAV vector expressing the fluorescent reporter CFP (AAV2/1- Ef1a-DIO-CFP). The extent of ablation was estimated *post hoc* by mapping the spatial extent of fluorophore expression from the AAV vector (Fig. 7*B*). The successful ablation of *Sox14*^+^ neurons in the IGL/LGv was further confirmed by ISH with an RNA probe against the *Npy* mRNA (Fig. 7*C*; reduction in *Npy*^+^ neurons: *Opn4*^*taulacZ*/+^;*Sox14*^*Cre*/+^ 88 ± 0.05%, *p* < 0.0001; *Opn4^taulacZ/taulacZ^*;*Sox14*^*Cre*/+^ 77 ± 0.14%, *p* = 0.0003; mean ± SEM; unpaired *t* test). DTA-ablated and control animals were single-housed in a circadian cabinet, and their spontaneous locomotor activity recorded via passive infrared detectors. Ablation of *Sox14*^+^ IGL/LGv neurons did not alter overall rhythmicity of motor behavior nor the circadian period length when animals were housed in constant darkness (tau; Fig. 7*D*); however, a trend toward increased period on IGL/LGv ablation was noted consistently with previous studies (Pickard, 1994). In both the melanopsin heterozygote (Fig. 7*E*,*G*) and knock-out background (Fig. 7*F*,*H*), ablation of the *Sox14*^+^ IGL/LGv neurons did not affect the ability of the mice to photoentrain to standard laboratory light conditions (200 lux;12 h light:12 h dark; Fig. 7*I*,*N*).

We then tested the ability of these animals to respond to a 6-h phase advance in the light cycle, maintaining all other conditions, including luminance, unchanged. Fourteen days after the light phase advance, all groups had entrained to the new light cycle (Fig. 7*I*,*O*), indicating that neither the *Sox14*^+^ IGL/LGv neurons nor melanopsin expression or both combined are required for circadian resetting of activity rhythms under standard luminance (200 lux). However, ablation of the *Sox14*^+^ IGL/LGv appeared to cause a delayed behavioral response to the light phase advance (Fig. 7*I*), which did not reach statistical significance after correction for multiple comparisons (Fig. 7*O*; minutes to re-entrainment at day 7: *Opn4*^*taulacZ*/+^*Sox14*-control 11.25 ± 11.25 min *Opn4*^*taulacZ*/+^*Sox14*-ablated: 104.4 ± 17.46 min; *Opn4^taulacZ/taulacZ^ Sox14*-control 43.75 ± 25.77 *Opn4^taulacZ/taulacZ^ Sox14*-ablated 119.2 ± 42.43 min; mean ± SEM). This observation of delayed entrainment under standard light conditions is consistent with earlier neurotoxin injections in the lateral geniculate of the hamster (Johnson et al., 1989).

We subsequently tested the ability of control and ablated mice to entrain to a further 6-h phase shift while simultaneously reducing the strength of the light zeitgeber to 10 lux; Fig. 7*J–N*). Luminance of 10 lux and below mimics the ecologically relevant twilight conditions and suffice to generate SCN activation and behavioral photoentrainment in laboratory mice (Cheng et al., 2004).

Regardless of the status of *Opn4* expression, by day 14 after the dim-light phase advance all but two of the animals that did not experience ablation of *Sox14*^+^ IGL/LGv neurons had successfully entrained their activity rhythms to the new dim light cycle (Fig. 7*O*; minutes to re-entrainment: *Opn4*^*taulacZ*/+^*Sox14*-control 30 ± 30 min; *Opn4^taulacZ/taulacZ^Sox14*-control 7.5 ± 7.5 min, mean ± SEM). Ablation of *Sox14*^+^ IGL/LGv impacted the ability of the animals to rapidly entrain to the new dim light cycle (Fig. 7*N*; *p* < 0.0001 Kruskal–Wallis test).

Ablation of the *Sox14*^+^ IGL/LGv had a differential effect on re-entrainment to the dim light cycle depending on the status of *Opn4* expression (Fig. 7*J–M*). By day 14 from the dim light phase advance, only three out of eight mice in the *Opn4*^*taulacZ*/+^*Sox14*-ablated group had entrained to the new light cycle and one animal displayed a lengthening of the circadian period (positive drifting; Fig. 7*O*). Furthermore, we observed irregular patters of activity onset (Fig. 7*L*) during the 14-d period, which were not present in the same animals exposed to the jet lag paradigm at 200 lux. Contrary to the result observed under 200 lux, under dim light the melanopsin loss of function accentuated the entrainment phenotype observed on ablation of the *Sox14*^+^ IGL/LGv (Fig. 7*N*) so that by day 14, none of the animals in the *Opn4^taulacZ/taulacZ^Sox14*-ablated group had entrained to the dim light cycle and three out of six animals displayed period lengthening (Fig. 7*M–O*; minutes to re-entrainment at day 7: *Opn4^taulacZ/taulacZ^Sox14*-control 122.5 ± 65.11 min, *Opn4^taulacZ/taulacZ^Sox14*-ablated: 450.0 ± 56.98 min, *p* = 0.0058, mean ± SEM, unpaired *t* test; minutes to re-entrainment at day 14: *Opn4^taulacZ/taulacZ^Sox14*-control 7.5 ± 7.5 min, *Opn4^taulacZ/taulacZ^Sox14*-ablated 480.8 ± 102.9 min, mean ± SEM, *p* = 0.0095, Mann–Whitney test).

Taken together, these data showed that while neither melanopsin expression nor the *Sox14*^+^ neurons of the IGL/LGv are required for entrainment and re-entrainment in a jet-lag paradigm, the *Sox14*^+^ IGL/LGv neurons contribute to the rapid resetting of circadian activity rhythms. Strikingly, while under luminance levels akin to twilight conditions melanopsin loss of function alone had little effect on the ability of the mice to photoentrain, the combined ablation of the *Sox14*^+^ IGL/LGv severely disrupted circadian photoentrainment of activity rhythms.

### Daily optogenetic stimulation of the *Sox14*^+^ IGL/LGv neurons entrains motor activity rhythms

Circadian rhythms of nocturnal animals can be entrained by pulses of light given at dusk and dawn, possibly reflective of the light sampling behavior displayed in their natural ecological niche (Rosenwasser et al., 1983; Stephan, 1983; DeCoursey, 1986; Edelstein and Amir, 1999). Consistent with those earlier studies, optogenetics-assisted resetting of circadian oscillatory activity in dark reared mice can be achieved by daily, 1 h-long, blue light pulses delivered at low frequency (4–8 Hz) on SCN neurons expressing ChR2 (Jones et al., 2015; Mazuski et al., 2018).

To investigate whether experimental activation of *Sox14*^+^ IGL/LGv neurons is also sufficient to alter circadian patterns of behavior, we aimed to replicate this artificial circadian entrainment protocol, stimulating the *Sox14*^+^ IGL/LGv neurons instead of the SCN ones. To achieve this, we injected either a Cre-dependent AAV vector expressing the light-gated ion channel Channelrodopsin2 (ChR2; AAV2/5 Ef1a-DIO-hChR2(H134R)-mCherry) or a control AAV expressing the cyan fluorescent protein (AAV2/1-Ef1a-DIO-CFP; Fig. 7*P*) bilaterally in the IGL/LGv region of *Sox14*^*Cre*/+^mice. We then tested the impact of forced ChR2-mediated activation of the *Sox14*^+^ IGL/LGv neurons for 1 h at daily intervals on the onset of circadian locomotor activity in animals housed under constant darkness (Fig. 7*P*).

Pulses of blue light (470 nm) were delivered bilaterally directly above the IGL/LGv at a frequency of 8 Hz (Fig. 7*P*), which falls within the physiological frequency range of IGL neurons (Lewandowski and Blasiak, 2004; Chrobok et al., 2018, 2021) and of the SCN neurons during the light phase of the day (Sakai, 2014; Jones et al., 2015). Furthermore, optogenetic 10-Hz stimulation of RGC axon terminals is sufficient to activate IGL neurons (Shi et al., 2020).

*Ex vivo* patch-clamp recording from ChR2-expressing IGL neurons confirmed reliable light-induced responses to 8 Hz blue light entrainment (Fig. 7*Q*). In vivo, this optogenetic protocol led to expression of the immediate early gene c-Fos in the IGL/LGv (Fig. 7*R*), which is a reliable marker of neuronal activation (Dragunow and Faull, 1989; Peters et al., 1996).

As expected, housing under constant dark conditions efficiently induced free running rhythms in control and experimental mice (Fig. 7*S–U*; white and black circles, respectively). However, while daily optogenetic light stimulation in control animals had no significant impact on the onset of circadian locomotor activity (Fig. 7*S*,*T*; white circles), it affected it profoundly in experimental animals that expressed ChR2 (Fig. 7*S*,*T*; black circles). The impact of daily stimulation of the *Sox14*^+^ IGL/LGv is reflected in the drastic change in the slope of the circadian onset of motor activity between the two groups (Fig. 7*U*; *p* = 0.0002; *t* test). The gradual shift of the activity onset, which moved progressively toward the time of stimulation and in some but not all cases, locked onto it, is strikingly similar to the results obtained when stimulation was applied directly onto the SCN (Jones et al., 2015; Mazuski et al., 2018).

Acute light pulses during the active phase in nocturnal animals caused negative masking of locomotor activity (Mrosovsky et al., 2001; Redlin, 2001; Morin and Studholme, 2014a), a response that has recently been suggested to depend on IGL neurons (Shi et al., 2020). In our experimental conditions, a reduction in spontaneous motor activity was detected during the first episode of optogenetic stimulation of the *Sox14*^+^ IGL/LGv neurons (motor activity change during optogenetic stimulation compared with hour following it ChR2 group: 38.51 ± 19.72%; CFP group: 391.4 ± 123.0%, mean ± SEM, *p* = 0.0093; *t* test); however, the decrease in motor activity was negligeable over seven consecutive days of optical stimulation (ChR2 group: 141.0 ± 13.76%; CFP group: 229.8 ± 37.48%, mean ± SEM, *p* = 0.036; *t* test).

The effect of the optogenetic stimulation of the *Sox14*^+^ IGL/LGv neurons on the phase of circadian motor activity was observed regardless of the lag time between the endogenous onset of locomotor activity and the time of the optogenetic stimulation (Fig. 7*S*, short lag time and Fig. 7*T*, long lag time). This optogenetically-induced effect on the onset of circadian motor activity is rapidly reversed on termination of the photostimulation (Fig. 7*S*,*T*). Hence, in absence of a strong zeitgeber such as circadian light, daily optogenetic stimulation of the *Sox14*^+^ neurons of the IGL/LGv is sufficient to reorganise circadian locomotor activity.

### The *Sox14*^+^ IGL/LGv neurons are required for rapid change in vigilance states at circadian light transitions

**Figure 8.**
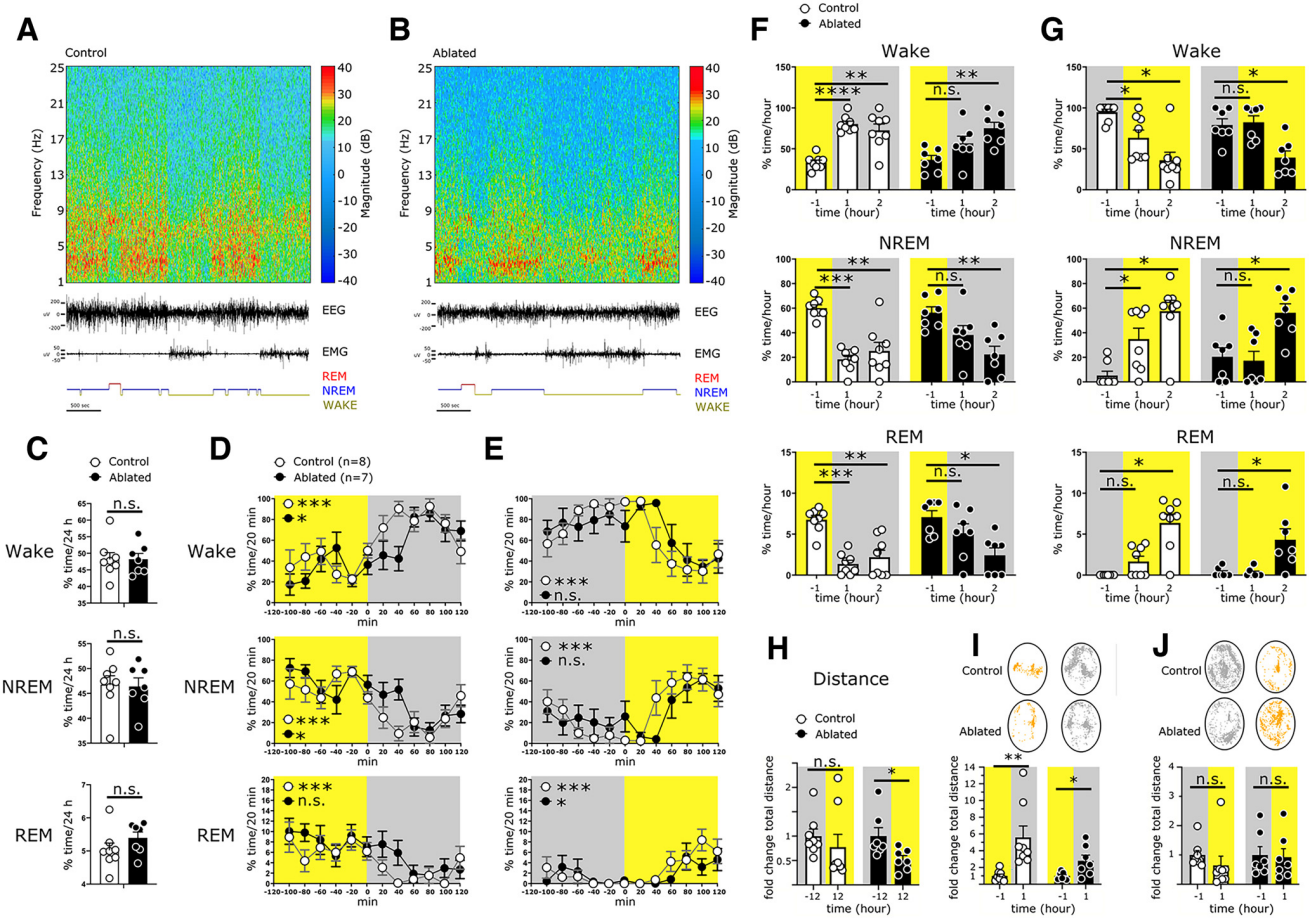
Ablation of *Sox14*^+^ IGL/LGv neurons causes delayed vigilance state transitions at circadian light changes. ***A***, ***B***, Spectrograms, EEG/EMG traces, and hypnograms over a 1-h period from a representative control mouse and an ablated mouse. ***C***, Over a 24-h period, control and *Sox14*^+^ IGL/LGv-ablated mice spend a similar percentage of time in Wake (48.24 ± 1.96% vs 48.22 ± 1.71%, *p* = 0.99), NREM (46.73 ± 1.80% vs 46.38 ± 1.71%, *p* = 0.89), and REM (5.03 ± 0.20% vs 5.39 ± 0.17%, *p* = 0.2). Values are mean ± SEM, *t* test. ***D***, ***E***, Distribution of Wake, NREM, and REM in the 2 h preceding and following each light transition. Light and dark hours are shaded in yellow and gray, respectively (see Extended Data Table 6-1 for comprehensive statistics). ***F***, Pairwise comparison in the content of Wake, NREM, and REM between the hour preceding and each of the 2 h following the light to dark transition. Control group: Wake_−1_: 33.17 ± 2.98%, Wake_1_: 80.26 ± 3.49%, *p* < 0.0001, Wake_2_: 72.61 ± 7.76%, *p* = 0.001; NREM_−1_: 60.08 ± 2.65%, NREM_1_: 18.39 ± 3.29%, *p* = 0.0001, NREM_2_: 25.17 ± 7.04%, *p* = 0.0015; REM_-1_: 6.75 ± 0.52%, REM_1_: 1.35 ± 0.45% *p* = 0.0004, REM_2_: 2.18 ± 0.85% *p* = 0.007. Ablated group: Wake_−1_ 36.57 ± 5.34%, Wake_1_: 56.67 ± 3.49%, *p* = 0.093, Wake_2_: 75.23 ± 7.24%, *p* = 0.008; NREM_−1_ 56.35 ± 4.73%, NREM_1_: 38.19 ± 7.68%, *p* = 0.093, NREM_2_: 22.36 ± 6.64%, *p* = 0.0081; REM_-1_: 7.08 ± 0.78%, REM_1_: 5.10 ± 1.16%, *p* = 0.296, REM_2_: 2.41 ± 0.90%, *p* = 0.031. Values are mean ± SEM, *t* test except Control REM_-1_ versus REM_2_, Ablated REM_-1_ versus REM_1_ and REM_2_ Wilcoxon test. ***G***, Pairwise comparison in the content of Wake, NREM, and REM between the hour preceding and each of the 2 h following the dark to light transition. Control group: Wake_−1_: 94.77 ± 3.48%, Wake_1_: 63.47 ± 9.42%, *p* = 0.015, Wake_2_: 36.11 ± 9.72%, *p* = 0.015; NREM_−1_: 5.22 ± 3.48%, NREM_1_: 34.87 ± 8.82% *p* = 0.015, NREM_2_: 57.51 ± 8.86% *p* = 0.015; REM_-1_: 0.00 ± 0.00%, REM_1_: 1.66 ± 0.64%, *p* = 0.125, REM_2_: 6.37 ± 1.09%, *p* = 0.015. Ablated group: Wake_−1_ 79.18 ± 7.51%, Wake_1_: 82.47 ± 7.80%, *p* = 0.812, Wake_2_: 39.37 ± 8.09%, *p* = 0.017; NREM_−1_ 20.50 ± 7.34%, NREM_1_: 17.25 ± 7.65%, *p* = 0.848, NREM_2_: 56.31 ± 7.22%, *p* = 0.021; REM_-1_: 0.31 ± 0.21%, REM_1_: 0.27 ± 0.20%, *p* > 0.999, REM_2_: 4.31 ± 1.34%, *p* = 0.0313. Values are mean ± SEM, Wilcoxon test except Ablated Wake_−1_ versus Wake_2_ and NREM_−1_ versus NREM_2_ *t* test. ***H***, Fold change in distance traveled in the light phase (12 h). Total distance in the dark phase (12 h) was set at 1. Control group: 1.0 ± 0.14 (dark) versus 0.77 ± 0.26 (light); Ablated group: 1.0 ± 0.17 (dark) versus 0.52 ± 0.82 (light). ***I***, Example trajectories for one control and one ablated mouse in the hour preceding and following the light change. Oval: ROI used for automated tracking; yellow: light, gray: dark. Histograms report the fold change in distance traveled in the hour preceding and following the light change. For the light to dark transition distance in the light was set at 1: Control group 1.0 ± 0.21 (light) 5.59 ± 1.36 (dark), *p* = 0.0078; Ablated group 1.0 ± 0.17 (light) 2.80 ± 0.68 (dark), *p* = 0.044. ***J***, For the dark to light transition distance in the dark was set at 1: Control group 1.0 ± 0.15 versus 0.64 ± 0.31, *p* = 0.148; Ablated group 1.0 ± 0.28 versus 0.92 ± 0.29, *p* = 0.468. Values are mean ± SEM, Wilcoxon test except for Ablated light to dark transition *t* test. N.s.: not statistically significant. A summary of statistical tests is available in Extended Data Table 8-1.

Circadian transitions between light and dark regulate neuronal network dynamics that contribute to shaping the sleep-wake cycle. We hypothesized that the broad innervation of the *Sox14*^+^ neurons in the IGL/LGv by visual networks may reflect an underappreciated role in regulating rapid changes in vigilance in response to circadian light transitions. Such brain network changes may not be readily detected by monitoring gross circadian locomotor activity but can be more reliably measured as changes in the spectral power of a cortical EEG. To monitor the impact of ablating the *Sox14*^+^ IGL/LGv neurons on the vigilance states of the brain at each light transition under standard circadian conditions, we injected male *Sox14*^*Cre*/+^ mice with either AAV Ef1a-mCherry-DIO-DTA (ablated group) or AAV Ef1a-DIO-CFP (control group) into the IGL/LGv region, replicating the genetic strategy previously described. Control and ablated mice were then implanted with screw type skull electrodes for EEG and stainless-steel wire type electrodes inserted into the trapezius muscle of the neck for EMG. During the EEG/EMG recording, animals could move freely in their cage. Both ablated and control animals displayed characteristic cortical spectrograms, EEG/EMG traces and hypnograms with detectable transitions between periods of high magnitude δ frequency oscillations and reduced mobility indicative of nonrapid eye movement (NREM) sleep (Fig. 8*A*,*B*), and high magnitude θ frequency oscillations occurring either without associated increase in the amplitude of the EMG signal, as is typical of REM sleep, or with associated EMG activity, as is typical of the wake state (Wake; Fig. 8*A*,*B*).

10.1523/JNEUROSCI.0112-21.2022.tab8-1 Extended Data Table 8-1 Statistical treatment of EEG/EMG data. Mean values, experimental error, and the parametric and nonparametric tests used to calculate statistical significance. F indicates Friedman test; W indicates Wilcoxon. Download Table 8-1, DOCX file.

Consistent with the pattern of circadian locomotion observed in the cohort of *Sox14*^+^ IGL/LGv-ablated mice tested for circadian light entrainment, the cumulative fraction of a 24-h period spent in NREM, REM, and Wake states did not differ between ablated and control groups (Fig. 8*C*; Extended Data Table 8-1; NREM: *p* = 0.89, REM: *p* = 0.2, Wake: *p* = 0.99), indicating that the *Sox14*^+^ neurons of the IGL/LGv are not required for sleep and wake overall. However, plotting the content of NREM, REM, and Wake for each hour of the circadian cycle revealed a discrepancy between ablated and control groups specifically in the hour following each light transition. We further increased the temporal resolution by binning NREM, REM, and Wake episodes in 20-min intervals for the 2 h preceding and following each light transition. This analysis revealed that, while both control and ablated animals increased the time spent in Wake in the first 2 h after lights off (Fig. 8*D*; Extended Data Table 8-1 for full statistical data; Wake Control: *p* < 0.0001; Wake Ablated: *p* = 0.015) and conversely, decreased it at lights on (Fig. 8*E*; Extended Data Table 8-1; Wake Control: *p* < 0.0001, Wake Ablated: *p* = 0.051), the ablated group remained for a protracted period in a state similar to the one preceding each light transition, responding with a delayed kinetic to the circadian light change (Fig. 8*D*,*E*).

To quantitatively assess the impact of circadian light transitions on Wake, NREM, and REM states on ablation of the *Sox14*^+^ IGL/LGv, we plotted the cumulative time spent in each of the three vigilance states during the hour preceding the light change (−1) and the first (1) and the second hour (2) after the light change for the control and ablated groups (Fig. 8*F*). In the control group, pairwise comparisons between the hour preceding and each of the 2 h following the light transition showed a strong and statistically significant change in each of the three vigilance states already in the first hour following the circadian light transition (Fig. 8*F*; Extended Data Table 8-1; Wake_−1_ vs Wake_1_: *p* < 0.0001; NREM_−1_ vs NREM_1_: *p* = 0.0001; REM_-1_ vs REM_1_: *p* = 0.0004). In contrast, change in the three vigilance states in the ablated group does not reach statistical significance during the first hour after the light transition (Fig. 8*F*; Extended Data Table 8-1; Wake_−1_ vs Wake_1_: *p* = 0.093; NREM_−1_ vs NREM_1_: *p* = 0.093; REM_−1_ vs REM_1_: *p* = 0.296). However, in the ablated group, change in Wake and NREM reached statistical significance in the second hour after the light transition (Fig. 8*F*; Extended Data Table 8-1; Wake_−1_ vs Wake_2_: *p* = 0.0080; NREM_−1_ vs NREM_2_: *p* = 0.0081), substantiating the interpretation that the *Sox14*^+^ IGL/LGv neurons are required for the rapid change in cortical network activity caused by circadian light transitions, but not for overall regulation of sleep and wake over a 24-h period.

We then investigated whether a similar requirement for the *Sox14*^+^ IGL/LGv neurons also exists at the dark to light transition. In the control group, the time spent in Wake, NREM, and REM over the 3-h period changed significantly (Extended Data Table 8-1 for full statistical data).

In keeping with the observed rapid changes in vigilance states at light to dark transition (Fig. 6*F*), rapid changes in Wake and NREM were also detected already in the first hour after the transition to lights-on (Fig. 8*G*; Extended Data Table 8-1; Wake_−1_ vs Wake_1_: *p* = 0.015; NREM_−1_ vs NREM_1_: *p* = 0.015; REM_−1_ vs REM_1_: *p* = 0.125). However, in the ablated group, significant changes in the three vigilance states only became apparent in the second hour after the light transition (Fig. 8*G*; Extended Data Table 8-1; Wake_−1_ vs Wake_2_: *p* = 0.017; NREM_−1_ vs NREM_2_: *p* = 0.021; REM_-1_ vs REM_2_: *p* = 0.031). Hence, *Sox14*^+^ IGL/LGv neurons are required at both circadian light transitions to elicit rapid changes in vigilance states.

While delayed responses to the circadian light transitions caused by the ablation of *Sox14*^+^ IGL/LGv neurons could readily be detected in the EEG/EMG data, we noted that they correlated less clearly with overt locomotor behavior. Change in the distance traveled before and after the circadian light transitions, assessed by automated video tracking (Pinnacle Technologies) of animals undergoing EEG/EMG recordings, did not reveal similarly striking changes within and between groups (Fig. 8*H–J*; Extended Data Table 8-1).

We therefore focused our analysis on cortical network activity rather than behavioral output and extended the analysis of the EEG data to quantify oscillatory activity in the δ (0.5–4 Hz), θ (6–9 Hz), and α (8–12 Hz) frequency ranges. High power δ oscillations are typically observed during NREM, while θ oscillations are associated with REM sleep as well as exploratory behavior and α oscillations are enriched during quiet wake.

**Figure 9.**
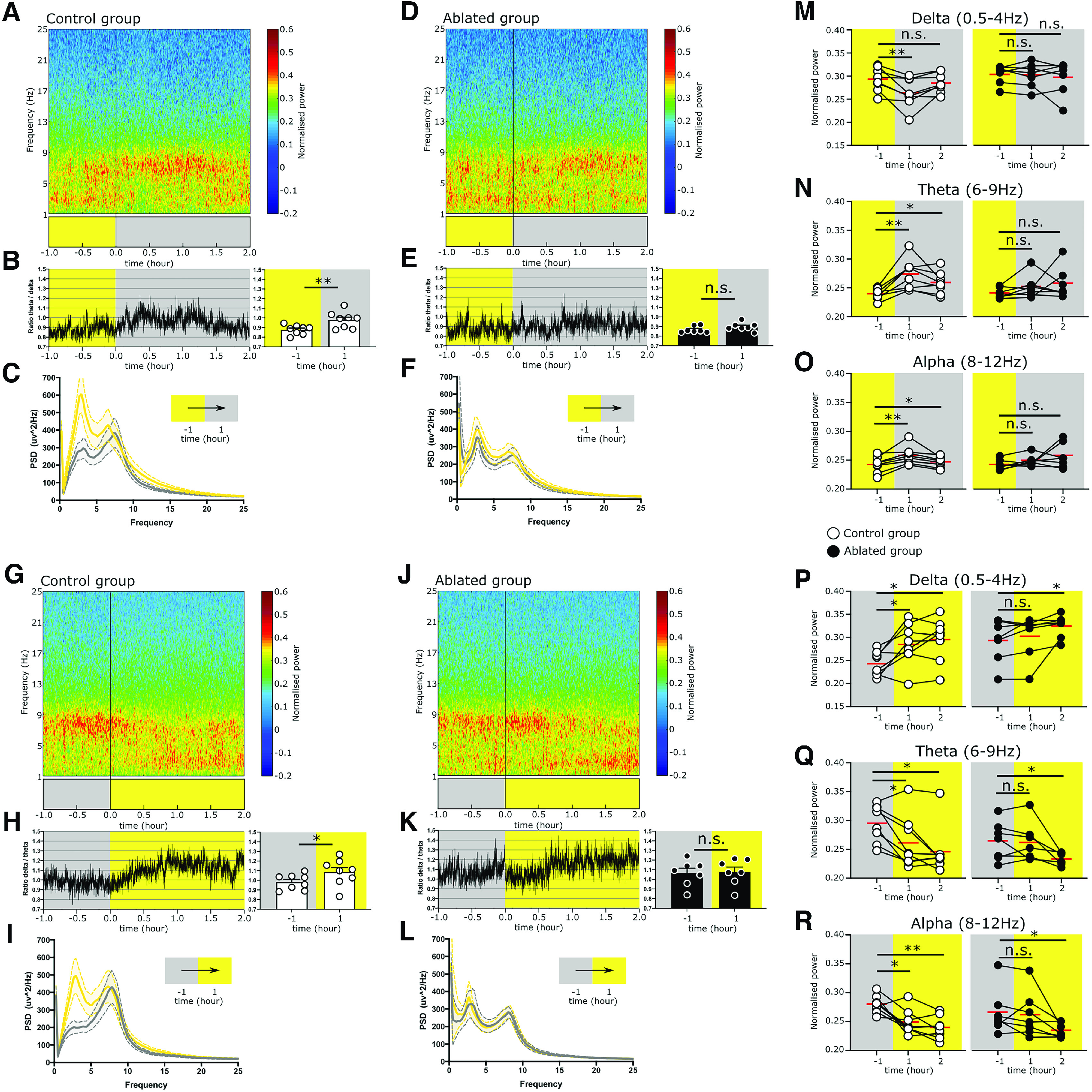
The *Sox14*^+^ IGL/LGv neurons are required for the rapid onset of cortical network activity associated with circadian light transitions. ***A***, Averaged cortical spectrogram for the Control group displaying a rapid increase in the θ power and decrease in δ power at the transition from lights on to lights off. ***B***, the change in θ and δ is displayed as a group average θ/δ ratio over time and as cumulative for the hour preceding and the hour following the light change. Control group (T/D)_−1_: 0.87 ± 0.01 versus (T/D)_1_: 0.97 ± 0.03, *p* = 0.001. Values are mean ± SEM, *t* test. ***C***, Power spectrum of the Control group in the hour preceding the transition from light to dark is overlayed to the power spectrum profile for the hour following the light transition (bold line: mean; dotted lines: ±SEM; yellow: light, gray: dark). ***D***, Averaged cortical spectrogram for the Ablated group displaying retention of high δ power following the lights on to lights off transition. ***E***, Change in θ and δ is displayed as a group average θ/δ ratio over time and as cumulative for the hour preceding and the hour following the light change. Ablated group (T/D)_−1_: 0.86 ± 0.01 versus (T/D)_1_: 0.89 ± 0.01, *p* = 0.078. Values are mean ± SEM, Wilcoxon test. ***F***, Power spectrum of the Ablated group in the hour preceding the transition from light to dark is overlayed to the power spectrum profile for the hour following the light transition (bold line: mean; dotted lines: ±SEM; yellow: light, gray: dark). ***G***, Averaged power spectrum for the Control group in the hour preceding and the 2 h following the lights off to lights on transition. ***H***, Change in the δ/θ ratio is plotted over time and as group mean for the hour preceding and following the light transition. Control group (D/T)_−1_: 0.98 ± 0.02 versus (D/T)_1_: 1.08 ± 0.04, *p* = 0.016. Values are mean ± SEM, *t* test. ***I***, Power spectrum of the Control group in the hour preceding the transition from dark to light overlayed to the power spectrum profile for the hour following the light transition (bold line: mean; dotted lines: ±SEM; yellow: light, gray: dark). ***J***, ***K***, ***L***, Same analysis as in G-H is applied to the Ablated group. Note that the Ablated group does not display a significative change in the δ/θ ratio. Ablated group (D/T)_−1_: 1.06 ± 0.05 versus (D/T)_1_: 1.07 ± 0.05, *p* = 0.623. Values are mean ± SEM, *t* test. ***M***, Pairwise comparison for the δ power for the hour preceding and each of the 2 h following the transition from lights on to lights off in the Control group (white circles) and Ablated group (filled black circles). Control group δ_–1_: 0.29 ± 0.009, δ_1_: 0.26 ± 0.01 *p* = 0.009, δ_2_: 0.28 ± 0.007 *p* = 0.323. Values are mean ± SEM, *t* test. Ablated group δ_–1_: 0.30 ± 0.007, δ_1_: 0.30 ± 0.009 *p* = 0.734, δ_2_: 0.29 ± 0.013 *p* = 0.937. Values are mean ± SEM, Wilcoxon test. ***N***, Pairwise comparison for the θ power for the hour preceding and each of the 2 h following the transition from lights on to lights off in the Control group (white circles) and Ablated group (filled black circles). Control group θ_–1_: 0.23 ± 0.003, θ_1_: 0.27 ± 0.009, *p* = 0.002, θ_2_: 0.25 ± 0.006, *p* = 0.105. Values are mean ± SEM, *t* test. Ablated group θ_–1_: 0.24 ± 0.003, θ_1_: 0.25 ± 0.007 *p* = 0.109, θ_2_: 0.25 ± 0.01, *p* = 0.109. Values are mean ± SEM, Wilcoxon test. ***O***, Pairwise comparison for the α power for the hour preceding and each of the 2 h following the transition from lights on to lights off in the Control group (white circles) and Ablated group (filled black circles). Control group α_–1_: 0.24 ± 0.004, α_1_: 0.25 ± 0.005, *p* = 0.003, α_2_: 0.24 ± 0.003, *p* = 0.045. Values are mean ± SEM, *t* test. Ablated group α_–1_: 0.24 ± 0.003, α_1_: 0.24 ± 0.003, *p* = 0.109, α_2_: 0.25 ± 0.008, *p* = 0.097. Values are mean ± SEM, Wilcoxon test. ***P***, ***R***, Similar pairwise analysis as in ***M–O*** but for the transition from lights off to lights on. Control group δ_–1_: 0.24 ± 0.009, δ_1_: 0.28 ± 0.016, *p* = 0.009, δ_2_: 0.29 ± 0.016, *p* = 0.019. Ablated group δ_–1_: 0.29 ± 0.017, δ_1_: 0.30 ± 0.017, *p* = 0.296, δ_2_: 0.32 ± 0.009, *p* = 0.036. Values are mean ± SEM, *t* test except for the Ablated group δ_2_ comparison which used a Wilcoxon test. Control group θ_–1_: 0.29 ± 0.011, θ_1_: 0.26 ± 0.016, *p* = 0.023, θ_2_: 0.24 ± 0.014, *p* = 0.023. Values are mean ± SEM, Wilcoxon test. Ablated group θ_–1_: 0.26 ± 0.012, θ_1_: 0.26 ± 0.012, *p* = 0.706, θ_2_: 0.23 ± 0.003, *p* = 0.038. Values are mean ± SEM, *t* test. Control group α_–1_: 0.27 ± 0.005, α_1_: 0.24 ± 0.007, *p* = 0.012, α_2_: 0.23 ± 0.006, *p* = 0.005. Values are mean ± SEM, *t* test. Ablated group α_–1_: 0.26 ± 0.014, α_1_: 0.26 ± 0.014, *p* = 0.375, α_2_: 0.23 ± 0.004, *p* = 0.046. Values are mean ± SEM, Wilcoxon test. N.s.: not statistically significant. A summary of statistical tests is available in Extended Data Table 9-1.

Power spectral densities (PSDs) displayed dynamic changes at either circadian light transition in both the control (Fig. 9*A,G*) and the ablated group (Fig. 9*D*,*J*). In the control group, time-frequency analysis of EEG data showed the presence of δ waves dominating the hour preceding the light to dark transition, with the appearance of θ and low α waves anticipating the light change and a sharp decrease in δ following the transition (Fig. 9*A*). The clear shift from δ to θ waves in the control group was supported by a θ/δ ratio (T/D) that was significantly increased after the circadian light change (Fig. 9*B*; Extended Data Table 9-1; (T/D)_−1_ vs (T/D)_1_ *p* = 0.0016) and the clear frequency shift from high δ and low θ state in the hour preceding the light transition (Fig. 9*C*, yellow line) to high θ and low δ state in the hour following the light transition (Fig. 9*C*, gray line).

10.1523/JNEUROSCI.0112-21.2022.tab9-1 Extended Data Table 9-1 Statistical treatment of EEG/EMG data. Mean values, experimental error, and the parametric and nonparametric tests used to calculate statistical significance. F indicates Friedman test; W indicates Wilcoxon. Download Table 9-1, DOCX file.

In stark contrast, the spectrogram of EEG data from the *Sox14*^+^ IGL/LGv ablated mice showed a mixture of δ, θ, and low-α waves in the hour before the light transition. δ waves continued to be present for the first hour after the light transition, eventually fading in the second hour, when θ and low-α oscillations increased (Fig. 9*D*). Consequently, there was no significant change in T/D ratio (Fig. 9*E*; (T/D)_−1_ vs (T/D)_1_: *p* = 0.078) and PSDs showed similar spectral patterns before (Fig. 7*F*, yellow line) and after the light transition (Fig. 9*F*, gray line). These observations are consistent with delayed cortical network dynamics at the lights-on to lights-off transition in mice with *Sox14*^+^ IGL/LGv ablation.

We then performed the same analysis for the lights-off to lights-on transition of the circadian day, which is normally accompanied by sleep onset. As expected, spectrograms from the control mice showed the presence of θ and low-α waves in the hour preceding the light transition, which shifted to high amplitude δ and reduced θ and low-α waves already within the first hour following the light transition (Fig. 9*G*). This clear shift from θ to δ waves in the control group was reflected in the increased δ/θ ratio (D/T; Fig. 9*H*; Extended Data Table 9-1; (D/T)_−1_ vs (D/T)_1_: *p* = 0.0168).

Similarly, PSD showed a clear frequency shift from high amplitude θ and low δ in the hour preceding the light transition (Fig. 9*I*, gray line) to high δ and low θ within the first hour following the light transition (Fig. 9*I*, yellow line).

The ablated mice differed from the control group as θ and low α waves persisted for ∼40 min following the light transition, after which they faded and were replaced by δ oscillations (Fig. 9*J*). Consequently, there was no significant change in D/T ratio in the hour preceding and following the light transition (Fig. 9*K*; (D/T)_−1_ vs (D/T)_1_: *p* = 0.623) and PSDs retained similar distribution before (Fig. 9*L*, gray line) and after the light transition (Fig. 9*L*, yellow line).

To assess quantitatively the change in power for the δ, θ, and α ranges, we calculated the cumulative power for the hour preceding and for the first and second hour following each circadian light transition, starting first from lights-on to lights-off. Across the three time points, animals in the control group showed significant change for all three frequency bands (Fig. 9*M–O*; δ *p* = 0.0064, θ *p* = 0.0012, α *p* = 0.0031). δ Power decreased significantly already in the first hour after the light transition (Fig. 9*M*; Extended Data Table 9-1; δ_–1_ vs δ_1_ *p* = 0.0099). Concomitantly, the θ power increased significantly (Fig. 9*N*; Extended Data Table 9-1; θ_–1_ vs θ_1_: *p* = 0.0027) as did α power (Fig. 9*O*; Extended Data Table 9-1; α_–1_ versus α_1_: *p* = 0.0039). In contrast, the group of animals with ablation of the *Sox14*^+^ neurons in the IGL/LGv did not display significant overall change in the power of δ, θ, and α across the three timepoints (Fig. 9*M–O*; Extended Data Table 9-1 for comprehensive statistical data).

We then performed the quantitative analysis on the combined hourly variation in δ, θ, and α for the second circadian light transition of the day, corresponding to lights off to lights on. As expected, significant difference was detected in the control group (δ: *p* = 0.011, θ: *p* = 0.030, α: *p* = 0.0029). Pair-wise comparisons between the hour preceding and each of the 2 h following the circadian light change confirmed a significant increase in δ power taking place already in the first hour (Fig. 9*P*; Extended Data Table 9-1; δ_–1_ vs δ_1_: *p* = 0.019), accompanied by a significant decrease in θ power (Fig. 9*Q*; Extended Data Table 9-1; θ_–1_ vs θ_1_: *p* = 0.023) and α power (Fig. 9*R*; Extended Data Table 9-1; α_–1_ vs α_1_: *p* = 0.0124).

In contrast, in the *Sox14*^+^ IGL/LGv ablated group, δ and α power did not change significantly across the three time points (Fig. 9*P*,*R*; Extended Data Table 9-1; δ: *p* = 0.051, α: *p* = 0.111). Power in the θ frequency across all time points showed a significant difference (Extended Data Table 9-1; θ: *p* = 0.028), which was due to a decrease in power in the second hour after the transition (Fig. 9*Q*; Extended Data Table 9-1; θ_–1_ vs θ_1_: *p* = 0.706, θ_–1_ vs θ_2_: *p* = 0.038).

In summary, power spectral analysis of cortical EEG reveals a previously undescribed role for the thalamic neurons in the IGL/LGv in enabling rapid changes in cortical network activity at both circadian light changes.

## Discussion

While the *Sox14*^+^ neurons constitute a thalamic cell class prominently found in the IGL, they also contribute to the prethalamic LGv nucleus. Several fate mapping experiments have demonstrated that LGv neurons arise from prethalamic progenitors (Vue et al., 2007; Delaunay et al., 2009; Inamura et al., 2011; Suzuki-Hirano et al., 2011; Golding et al., 2014; Puelles et al., 2020). Here, we have shown that prethalamic progenitors make a significative contribution to the IGL, a structure of the thalamus proper. The specific circuit organization of the developmentally defined lineages that make up the IGL/LGv complex is largely unknown. Our monosynaptic restricted retrograde tracing places the *Sox14*^+^ IGL/LGv firmly within the visual system, with input from cortical and subcortical visual structures. Innervation of the *Sox14*^+^ neurons from the cholinergic, monoaminergic and orexinergic neurons appeared more limited, contrasting with previous classic tract tracing experiments of the anatomically defined IGL/LGv complex (Morin, 2013). It would be intriguing to test whether input to prethalamic lineages of the IGL/LGv displayed a complementary shift toward the ascending arousal system.

Previous anterograde tracing of retinal input from *Opn4*^+^ RGCs has provided clear evidence of enriched innervation of the IGL/LGv from multiple ipRGC types (Chen et al., 2011) as well as conventional RGC types (Beier et al., 2020). Tracing with an M1-enriched reporter construct identified the IGL, which receives afferents from both the *Brn3b*^+^ and the *Brn3b^neg^* ipRGCs (Chen et al., 2011), as a major target of the M1 ipRGC subtype (Hattar et al., 2006; Ecker et al., 2010). Surprisingly, our monosynaptic retrograde tracing from *Sox14*^+^ IGL/LGv did not label any M1 ipRGCs, but highlighted input from RGC types that express low levels of *Opn4* and may be more reliant on classic photoreceptors for luminance detection than the M1 type (Sonoda and Schmidt, 2016). As the vast majority of ipRGCs projecting to the SCN are the *Brn3b^neg^* M1 type (Baver et al., 2008; Chen et al., 2011), which also participate in innervation of the IGL/LGv (Chen et al., 2011), it is likely that some neurons in the IGL/LGv receive a copy of the SCN's retinal input (Pickard, 1985), however, here we show that the *Sox14*^+^ neurons of the IGL/LGv complex do not seem to participate in such M1 driven circuitry suggesting that other developmental lineages, potentially of the prethalamic *Dlx5/6*^+^ type, could display such specific retinal input. Alternatively, M1 ipRGC input to *Sox14*^+^ IGL/LGv neurons may involve a rare cell subtype and hence not have been detected in our samples. Given the reliance of our investigation on the transsynaptic retrograde properties of the SADB19 rabies strain, we have sought to prove in control experiments that it can infect the M1-ipRGCs. However, it should also be considered that differential efficiencies in retrograde transport and cytotoxic effects could potentially contribute to distort the relative abundance of selected retinal afferents.

Intact retinohypothalamic connectivity and preservation of a hypothetical M1 ipRGC-driven IGL/LGv tract to the SCN would ensure circadian photoentrainment upon ablation of the *Sox14*^+^ IGL/LGv, unless the strength of the photic cue is reduced, as observed in our experimental conditions. Our findings are consistent with the hypothesis put forward by Pickard that neurons of the geniculohypothalamic tract may convey information on illumination intensity to the SCN (Pickard et al., 1987). The connectional bias of the *Sox14*^+^ IGL/LGv toward subcortical and cortical visual networks bears implications for the view that integration of photic and nonphotic circadian cues takes place in IGL/LGv neurons to provide a unified output to the SCN. Our data could also be compatible with the presence of segregated pathways to the SCN via different subtypes of IGL/LGv neurons.

Under circadian dim light, the observed high interindividual variability of the activity onset in *Sox14*^+^ IGL/LGv-ablated mice with or without melanopsin expression, may be explained by the unmasking of one or more *Sox14^neg^* circuitries for internal state-dependent modulation of the circadian clock.

The use of dim light of amplitudes comparable to dusk and dawn carries ethological value, as nocturnal animals in their natural environment are more likely to sample light from a dark burrow and such transient exposure suffice in providing photoentrainment. Indeed, studies on nocturnal flying squirrels (*Glaucomys volans)* and other rodents that made use of a den cage, have shown that in nocturnal animals the light sampling behavior appears to be under circadian control, at dusk and dawn (Twente, 1955; Pratt and Goldman, 1986). It is likely that in their ecological niche nocturnal animals are exposed to just few minutes of light each day (DeCoursey, 1986). Accordingly, regular pulses of light at dusk and dawn are sufficient to photoentrain behavioral rhythms in nocturnal animals (DeCoursey, 1972; Rosenwasser et al., 1983; Stephan, 1983). Here, we have shown, using an optogenetics strategy, that repeated circadian activation of *Sox14*^+^ neurons in the IGL/LGv interrupts the spontaneous drifting of endogenous activity rhythms in mice kept under constant darkness and results in entrainment of the activity onset, in striking similarity to the effect caused by an analog optogenetics stimulation of the SCN (Jones et al., 2015; Mazuski et al., 2018).

In nocturnal rodents, acute light exposure causes negative masking of motor activity, a process that involves rapid NREM induction (Mrosovsky et al., 2001; Lupi et al., 2008; Morin and Studholme, 2009, 2014a). The subcortical networks involved in this response to acute light exposure are not fully mapped but are thought to involve the pretectum and superior colliculus (Miller et al., 1998; Zhang et al., 2019). More recently, a genetic strategy to ablate GABAergic neurons in the IGL region resulted in reduced NREM sleep on acute light presentation in mice (Shi et al., 2020), implicating the IGL/LGv complex as an important node in the phenomenon of photosomnolence. It remained unclear whether the IGL/LGv is also required for vigilance state changes at recurrent and predictable circadian light transitions.

IpRGCs, with their connectivity to the SCN, but also directly to other brain regions, play an important role in the control of sleep and arousal (Altimus et al., 2008; Lupi et al., 2008; Tsai et al., 2009; Muindi et al., 2013; Pilorz et al., 2016; Rupp et al., 2019). Circadian light transitions initiate a cascade of events that likely involves multiple brain networks and results in the stabilization of a new vigilance state. Here, we show that the thalamic *Sox14*^+^ neurons of the IGL/LGv ensure rapid transition to NREM-associated δ frequency cortical oscillations at lights-on and, conversely, the rapid establishment of a wake cortical profile at lights-off. Our data substantiate and expand earlier speculative hypotheses implicating the IGL in sleep regulation (Horowitz et al., 2004; Morin and Blanchard, 2005; Morin, 2013, 2015). However, the precise downstream events that are elicited by *Sox14*^+^ IGL/LGv neurons at circadian light transitions remain to be fully elucidated.

Defective encoding of circadian light changes emerges as one of the themes from the cell ablation approach presented here. This is consistent with the failure to photoentrain circadian activity rhythms described in the constitutive *Sox14* knock-out mice (Delogu et al., 2012); however, while ablation of *Sox14*^+^ IGL/LGv neurons in the mature brain caused delayed transitions in vigilance states at circadian light changes, defective photoentrainment of locomotor activity was only detectable under reduced strength of the lighting cues. It is likely that *Sox14* loss of function during brain development across neurons of the subcortical visual shell causes widespread changes in network connectivity and neuronal function that result in more overt changes in arousal and circadian behaviors.

By taking a developmentally informed approach, we have identified specific contributions of thalamic neurons of the IGL and LGv that expand our understanding of thalamic function in sensory perception and the control of vigilance states. While this approach reduced the developmental complexity of the anatomically defined IGL/LGv to reveal some of its unique connectional and functional properties, it does not resolve the further differentiation of the *Sox14*^+^ developmental lineage into subsets of molecularly defined mature neurons. Future investigations that exploit single cell genomics and connectomics will reveal the finer grain of parallel and integrated pathways occurring on thalamic projection GABAergic neurons.
