# Supplementary material for: A Role for Thalamic Projection GABAergic Neurons in Circadian Responses to Light
Source: J Neurosci. 2022 Dec 7;42(49):9158–79. doi: 10.1523/JNEUROSCI.0112-21.2022 (PMC9761691; doi:10.1523/JNEUROSCI.0112-21.2022)
Supplement: Extended Data Table 6-1 — Anatomical classification of regions harboring presynaptic input to Sox14+ neurons. The classification used to cluster anatomical regions containing cells transsynaptically labeled by the RVdG vector is based on the atlas of the mouse brain by Paxinos and Franklin (Paxinos and Franklin, 2001). Download Table 6-1, DOCX file. [file ns-JN-RM-0112-21-s01.docx]

| **Anterior pretectum** | **Oculomotor** |
| --- | --- |
| anterior pretectal nucleus, bilateral | oculomotor nucleus |
| **Arousal system** | nucleus of Darkschewitsch |
| dorsomedial tegmental area | dorsal terminal nucleus of the accessory optic tract |
| dorsal raphe nucleus | Edinger-Westphal nucleus |
| locus coeruleus, bilateral | interstitial nucleus of Cajal |
| laterodorsal tegmental nucleus, bilateral | medial accessory oculomotor nucleus |
| median raphe nucleus | visual tegmental relay zone |
| periacqueductal grey | **Optic tract** |
| parabrachial nuclei, bilateral | **Other cortices** |
| paramedian raphe nucleus | primary auditory cortex |
| peduncolopontine tegmentum, bilateral | secondary auditory cortex |
| rostral linear nucleus of the raphe | temporal association cortex |
| raphe magnus nucleus | claustrum |
| ventrolateral periacqueductal gray | dorsal endopiriform nucleus |
| **Basal ganglia** | dorsal peduncular cortex |
| bed nucleus of stria terminalis | ecthorinal cortex |
| caudate putamen | lateral orbital cortex |
| globus pallidus | lateral parietal association cortex |
| substantia nigra | primary motor cortex |
| ventral tegmental area | secondary motor cortex |
| **Hypothalamus** | piriform cortex |
| anterior hypothalamus | primary somatosensory cortex |
| dorsomedial hypothalamus | secondary somatosensory coretx |
| lateral hypothalamus, bilateral | **Other/unclassified** |
| medial hypothalamus, bilateral | cerebral peduncle, basal part |
| posterior hypothalamus, bilateral | mamillotegmental tract |
| parasubthalamic nucleus | nigrostriatal bundle |
| paraventricular hypothalamic nucleus | peripeduncular nucleus |
| ventromedial hypothalamus | **Pontine region** |
| **Inferior colliculus** | pontine nucleus, bilateral |
| dorsal cortex of the inferior colliculus, bilateral | **Posterior commissural area** |
| external cortex of the inferior colliculus, bilateral | retroparafascicular nucleus |
| **Limbic system** | nucleus of the posterior commissure |
| anterodorsal thalamic nucleus | precommissural nucleus, bilateral |
| amygdala | subcommissural nucleus |
| cingulate cortex | **Prethalamus** |
| dentate gyrus | reticular thalamic nucleus |
| infralimbic cortex | zona incerta |
| laterodorsal thalamic nucleus | **Retina** |
| lateral habenular nucleus | retina, bilateral |
| lateral septal nucleus | **Subcortical visual shell** |
| mammillary bodies | intergeniculate leaflet, bilateral |
| medial habenular nucleus | dorsolateral geniculate nucleus |
| medial orbital cortex | ventrolateral geniculate nucleus, bilateral |
| prelimbic cortex | nucleus of the optic tract |
| paratenial thalamic nucleus | olivary pretectal nucleus |
| paraventricular thalamic nucleus | medial pretectal nucleus |
| reuniens thalamic nucleus | posterior pretectal nucleus, bilateral |
| retrosplenial cortex | optic nerve layer of the superior colliculus, bilateral |
| submedius thalamic nucleus | parabigeminal nucleus |
| nuclei of the diagonal band | perihabenular nucleus, bilateral |
| **Medulla** | posterior limitans thalamic nucleus |
| external cuneate nucleus | suprachiasmatic nucleus |
| gigantocellular reticular nucleus | subgeniculate nucleus |
| medial vestibular nucleus | superficial gray layer of the superior colliculus, bilateral |
| superior vestibular nucleus | zonal layer of the superior colliculus |
| superior cerebellar peduncule | **Superior colliculus deep layers** |
| **Midbrain reticular formation** | deep layers of the superior colliculus |
| cuneiform nucleus, bilateral | intermediate layers of the superior colliculus, bilateral |
| deep mesencephalic nucleus, bilateral | **Thalamus** |
| midbrain reticular nucleus | lateroposterior thalamic nucleus |
| rostral interstitial nucleus of medial longitudinal fasciculus | medial geniculate nucleus |
| **Midbrain tegmentum** | parafascicular thalamic nucleus |
| prerubral field | posterior intralaminar thalamic nucleus bilateral |
| red nucleus | subparafascicular thalamic nucleus |
| sagulum nucleus | ventromedial thalamic nucleus |
|  | **Visual cortex** |
|  | secondary visual cortex |
|  | primary visual cortex |
