# Supplementary material for: A Role for Thalamic Projection GABAergic Neurons in Circadian Responses to Light
Source: J Neurosci. 2022 Dec 7;42(49):9158–79. doi: 10.1523/JNEUROSCI.0112-21.2022 (PMC9761691; doi:10.1523/JNEUROSCI.0112-21.2022)
Supplement: Extended Data Table 8-1 — Statistical treatment of EEG/EMG data. Mean values, experimental error, and the parametric and nonparametric tests used to calculate statistical significance. F indicates Friedman test; W indicates Wilcoxon. Download Table 8-1, DOCX file. [file ns-JN-RM-0112-21-s02.docx]

| **Parameter** | **Mean ± s.e.m.** | **t/Wilcoxon (W) statistics** | **RM ANOVA /Friedman (F) statistics** | **p value** | **Fig. n.** |
| --- | --- | --- | --- | --- | --- |
| **24 hours period** |  |  |  |  |  |
| Total Wake (Control vs Ablated) | 48.24 ± 1.96 vs 48.22 ± 1.71 | 0.0072 | N/A | 0.99 | 4C |
| Total NREM (Control vs Ablated) | 46.73 ± 1.80 vs 46.38 ± 1.71 | 0.139 | N/A | 0.89 | 4C |
| Total REM (Control vs Ablated) | 5.03 ± 0.20 vs 5.39 ± 0.17 | 1.326 | N/A | 0.2 | 4C |
| **2 hours before and 2 hours after the light to dark transition** | | |  |  |  |
| Wake L/D Control | N/A | N/A | 24.2 | ***<0.0001*** | 4D |
| Wake L/D Ablated | N/A | N/A | 6.444 | ***0.015*** | 4D |
| NREM L/D Control | N/A | N/A | 22.46 | ***0.0001*** | 4D |
| NREM L/D Ablated | N/A | N/A | 6.092 | ***0.018*** | 4D |
| REM L/D Control | N/A | N/A | 12.45 (F) | ***0.0005*** | 4D |
| REM L/D Ablated | N/A | N/A | 4.66 (F) | 0.099 | 4D |
| **2 hours before and 2 hours after the dark to light transition** | | |  |  |  |
| Wake D/L Control | N/A | N/A | 14 (F) | ***<0.0001*** | 4E |
| Wake D/L Ablated | N/A | N/A | 5.85 (F) | 0.051 | 4E |
| NREM D/L Control | N/A | N/A | 14 (F) | ***<0.0001*** | 4E |
| NREM D/L Ablated | N/A | N/A | 4.84 (F) | 0.09 | 4E |
| REM D/L Control | N/A | N/A | 12.25 (F) | ***0.0003*** | 4E |
| REM D/L Ablated | N/A | N/A | 8.27 (F) | ***0.019*** | 4E |
| **1 hour before compared to the first or second hour after the light to dark transition** | | | | | |
| Wake_Control_L(-1) vs D(+1) | 33.17 ± 2.98 vs 80.26 ± 3.49 | 8.066 | N/A | ***<0.0001*** | 4F |
| Wake_Control_L(-1) vs D(+2) | 33.17 ± 2.98 vs 72.61 ± 7.76 | 5.366 | N/A | ***0.001*** | 4F |
| Wake_Ablated_L(-1) vs D(+1) | 36.57 ± 5.34 vs 56.67 ± 3.49 | 1.992 | N/A | 0.093 | 4F |
| Wake_Ablated_L(-1) vs D(+2) | 36.57 ± 5.34 vs 75.23 ± 7.24 | 3.899 | N/A | ***0.008*** | 4F |
| NREM_Control_L(-1) vs D(+1) | 60.08 ± 2.65 vs 18.39 ± 3.29 | 7.768 | N/A | ***0.0001*** | 4F |
| NREM_Control_L(-1) vs D(+2) | 60.08 ± 2.65 vs 25.17 ± 7.04 | 5.051 | N/A | ***0.0015*** | 4F |
| NREM_Ablated_L(-1) vs D(+1) | 56.35 ± 4.73 vs 38.19 ± 7.68 | 1.992 | N/A | 0.093 | 4F |
| NREM_Ablated_L(-1) vs D(+2) | 56.35 ± 4.73 vs 22.36 ± 6.64 | 3.89 | N/A | ***0.0081*** | 4F |
| REM_Control_L(-1) vs D(+1) | 6.75 ± 0.52 vs 1.35 ± 0.45 | 6.363 | N/A | ***0.0004*** | 4F |
| REM_Control_L(-1) vs D(+2) | 6.75 ± 0.52 vs 2.18 ± 0.85 | -36 (W) | N/A | ***0.007*** | 4F |
| REM_Ablated_L(-1) vs D(+1) | 7.08 ± 0.78 vs 5.10 ± 1.16 | -14 (W) | N/A | 0.296 | 4F |
| REM_Ablated_L(-1) vs D(+2) | 7.08 ± 0.78 vs 2.41 ± 0.90 | -26 (W) | N/A | ***0.031*** | 4F |
| **1 hour before compared to the first or second hour after the dark to light transition** | | | | | |
| Wake_Control_D(-1) vs L(+1) | 94.77 ± 3.48 vs 63.47 ± 9.42 | -28 (W) | N/A | ***0.015*** | 4G |
| Wake_Control_D(-1) vs L(+2) | 94.77 ± 3.48 vs 36.11 ± 9.72 | -28 (W) | N/A | ***0.015*** | 4G |
| Wake_Ablated_D(-1) vs L(+1) | 79.18 ± 7.51 vs 82.47 ± 7.80 | 3 (W) | N/A | 0.812 | 4G |
| Wake_Ablated_D(-1) vs L(+2) | 79.18 ± 7.51 vs 39.37 ± 8.09 | 3.254 | N/A | ***0.017*** | 4G |
| NREM_Control_D(-1) vs L(+1) | 5.22 ± 3.48 vs 34.87 ± 8.82 | 28 (W) | N/A | ***0.015*** | 4G |
| NREM_Control_D(-1) vs L(+2) | 5.22 ± 3.48 vs 57.51 ± 8.86 | 28 (W) | N/A | ***0.015*** | 4G |
| NREM_Ablated_D(-1) vs L(+1) | 20.50 ± 7.34 vs 17.25 ± 7.65 | -3 (W) | N/A | 0.848 | 4G |
| NREM_Ablated_D(-1) vs L(+2) | 20.50 ± 7.34 vs 56.31 ± 7.22 | 3.078 | N/A | ***0.021*** | 4G |
| REM_Control_D(-1) vs L(+1) | 0.00 ± 0.00 vs 1.66 ± 0.64 | 10 (W) | N/A | 0.125 | 4G |
| REM_Control_D(-1) vs L(+2) | 0.00 ± 0.00 vs 6.37 ± 1.09 | 28 (W) | N/A | ***0.015*** | 4G |
| REM_Ablated_D(-1) vs L(+1) | 0.31 ± 0.21 vs 0.27 ± 0.20 | -1 (W) | N/A | >0.999 | 4G |
| REM_Ablated_D(-1) vs L(+2) | 0.31 ± 0.21 vs 4.31 ± 1.34 | 26 (W) | N/A | ***0.0313*** | 4G |
| **24 hours period** |  |  |  |  |  |
| Total Distance _12hr_D vs L_Control | 100 ± 14.54 vs 77.49 ± 26.22 | -18 (W) | N/A | 0.25 | 4H |
| Total Distance _12hr_D vs L_Ablated | 100 ± 17.38 vs 51.82 ± 8.19 | -26 (W) | N/A | ***0.031*** | 4H |
| **1 hour before and 1 hour after the light to dark transition** | | |  |  |  |
| Total Distance _One Hour_L vs D_Control | 100 ± 21.26 vs 559.3 ± 136.2 | 36 (W) | N/A | ***0.0078*** | 4I |
| Total Distance _One Hour_L vsD_Ablated | 100 ± 16.65 vs 279.9 ± 67.93 | 2.539 | N/A | ***0.044*** | 4I |
| **1 hour before and 1 hour after the dark to light transition** | | |  |  |  |
| Total Distance _One Hour_D vs L_Control | 100 ± 15.13 vs 64.49 ± 31.44 | -22(W) | N/A | 0.148 | 4J |
| Total Distance _One Hour_D vs L_Ablated | 100 ± 28.52 vs 92.37 ± 29.29 | -10(W) | N/A | 0.468 | 4J |
