# Supplementary material for: A Role for Thalamic Projection GABAergic Neurons in Circadian Responses to Light
Source: J Neurosci. 2022 Dec 7;42(49):9158–79. doi: 10.1523/JNEUROSCI.0112-21.2022 (PMC9761691; doi:10.1523/JNEUROSCI.0112-21.2022)
Supplement: Extended Data Table 9-1 — Statistical treatment of EEG/EMG data. Mean values, experimental error, and the parametric and nonparametric tests used to calculate statistical significance. F indicates Friedman test; W indicates Wilcoxon. Download Table 9-1, DOCX file. [file ns-JN-RM-0112-21-s03.docx]

| **Parameter** | **Mean ± s.e.m.** | **t /Wilcoxon (W) statistics** | **RM ANOVA/ Friedman (F) statistics** | **p value** | **Fig. n.** |
| --- | --- | --- | --- | --- | --- |
| **1 hour before and 1 hour after the light to dark transition** | | |  |  |  |
| Theta/Delta_Control_L(-1) vs D(+1) | 0.87 ± 0.01 vs 0.97 ± 0.03 | 4.996 | N/A | ***0.001*** | 5B |
| Theta/Delta_Ablated_L(-1) vs D(+1) | 0.86 ± 0.01 vs 0.89 ± 0.01 | 22 (W) | N/A | 0.078 | 5E |
| **1 hour before and 1 hour after the dark to light transition** | | |  |  |  |
| Theta/Delta_Control_D(-1) vs L(+1) | 0.98 ± 0.02 vs 1.08 ± 0.04 | 3.123 | N/A | ***0.016*** | 5H |
| Theta/Delta_Ablated_D(-1) vs L(+1) | 1.06 ± 0.05 vs 1.07 ± 0.05 | 0.517 | N/A | 0.623 | 5K |
| **1 hour before compared to the first or second hour after the light to dark transition** | | | | | |
| Delta_Control_Light/Dark | N/A | N/A | 7.672 | ***0.006*** | 5M |
| Delta_Ablated_Light/Dark | N/A | N/A | 0.857(F) | 0.768 | 5M |
| Delta_Control_L(-1) vs D(+1) | 0.29 ± 0.009 vs 0.26 ± 0.01 | 3.509 | N/A | ***0.009*** | 5M |
| Delta_Control_L(-1) vs D(+2) | 0.29 ± 0.009 vs 0.28 ± 0.007 | 1.063 | N/A | 0.323 | 5M |
| Delta_Ablated_L(-1) vs D(+1) | 0.30 ± 0.007 vs 0.30 ± 0.009 | -5(W) | N/A | 0.734 | 5M |
| Delta_Ablated_L(-1) vs D(+2) | 0.30 ± 0.007 vs 0.29 ± 0.013 | -2(W) | N/A | 0.937 | 5M |
| **1 hour before compared to the first or second hour after the light to dark transition** | | | | | |
| Theta_Control_Light/Dark | N/A | N/A | 12.02 | ***0.001*** | 5N |
| Theta_Ablated_Light/Dark | N/A | N/A | 2(F) | 0.486 | 5N |
| Theta_Control_L(-1) vs D(+1) | 0.23 ± 0.003 vs 0.27± 0.009 | 4.535 | N/A | ***0.002*** | 5N |
| Theta_Control_L(-1) vs D(+2) | 0.23 ± 0.003 vs 0.25± 0.006 | 3.2 | N/A | ***0.105*** | 5N |
| Theta_Ablated_L(-1) vs D(+1) | 0.24 ± 0.003 vs 0.25± 0.007 | 20(W) | N/A | 0.109 | 5N |
| Theta_Ablated_L(-1) vs D(+2) | 0.24 ± 0.003 vs 0.25± 0.01 | 20(W) | N/A | 0.109 | 5N |
| **1 hour before compared to the first or second hour after the light to dark transition** | | | | | |
| Alpha_Control_Light/Dark | N/A | N/A | 13.31 | ***<0.0001*** | 5O |
| Alpha_Ablated_Light/Dark | N/A | N/A | 2(F) | 0.486 | 5O |
| Alpha_Control_L(-1) vs D(+1) | 0.24 ± 0.004 vs 0.25± 0.005 | 4.231 | N/A | ***0.003*** | 5O |
| Alpha_Control_L(-1) vs D(+2) | 0.24 ± 0.004 vs 0.24± 0.003 | 2.429 | N/A | ***0.045*** | 5O |
| Alpha_Ablated_L(-1) vs D(+1) | 0.24 ± 0.003 vs 0.24± 0.003 | 20(W) | N/A | 0.109 | 5O |
| Alpha_Ablated_L(-1) vs D(+2) | 0.24 ± 0.003 vs 0.25± 0.008 | 1.964 | N/A | 0.097 | 5O |
| **1 hour before compared to the first or second hour after the dark to light transition** | | | | | |
| Delta_Control_Dark/Light | N/A | N/A | 8.42 | ***0.011*** | 5P |
| Delta_Ablated_Dark/Light | N/A | N/A | 6(F) | 0.051 | 5P |
| Delta_Control_D(-1) vs L(+1) | 0.24 ± 0.009 vs 0.28± 0.016 | 3.036 | N/A | ***0.019*** | 5P |
| Delta_Control_D(-1) vs L(+2) | 0.24 ± 0.009 vs 0.29± 0.016 | 3.13 | N/A | ***0.016*** | 5P |
| Delta_Ablated_D(-1) vs L(+1) | 0.29 ± 0.017 vs 0.30± 0.017 | 14(W) | N/A | 0.296 | 5P |
| Delta_Ablated_D(-1) vs L(+2) | 0.29 ± 0.017 vs 0.32± 0.009 | 2.684 | N/A | ***0.036*** | 5P |
| **1 hour before compared to the first or second hour after the dark to light transition** | | | | | |
| Theta_Control_Dark/Light | N/A | N/A | 7(F) | ***0.03*** | 5Q |
| Theta_Ablated_Dark/Light | N/A | N/A | 5.894 | ***0.028*** | 5Q |
| Theta_Control_D(-1) vs L(+1) | 0.29 ± 0.011 vs 0.26± 0.016 | -32(W) | N/A | ***0.023*** | 5Q |
| Theta_Control_D(-1) vs L(+2) | 0.29 ± 0.011 vs 0.24± 0.014 | -32(W) | N/A | ***0.023*** | 5Q |
| Theta_Ablated_D(-1) vs L(+1) | 0.26 ± 0.012 vs 0.26± 0.012 | 0.396 | N/A | 0.706 | 5Q |
| Theta_Ablated_D(-1) vs L(+2) | 0.26 ± 0.012 vs 0.23± 0.003 | 2.648 | N/A | ***0.038*** | 5Q |
| **1 hour before compared to the first or second hour after the dark to light transition** | | | | | |
| Alpha_Control_Dark/Light | N/A | N/A | 12.01 | ***0.002*** | 5R |
| Alpha_Ablated_Dark/Light | N/A | N/A | 4.571(F) | 0.111 | 5R |
| Alpha_Control_D(-1) vs L(+1) | 0.27 ± 0.005 vs 0.24± 0.007 | 3.344 | N/A | ***0.012*** | 5R |
| Alpha_Control_D(-1) vs L(+2) | 0.27 ± 0.005 vs 0.23± 0.006 | 3.975 | N/A | ***0.005*** | 5R |
| Alpha_Ablated_D(-1) vs L(+1) | 0.26 ± 0.014 vs 0.26± 0.014 | -12(W) | N/A | 0.375 | 5R |
| Alpha_Ablated_D(-1) vs L(+2) | 0.26 ± 0.014 vs 0.23± 0.004 | -24(W) | N/A | 0.046 | 5R |
